# Integrated co-expression analysis of host–parasite transcriptomes reveals mechanisms of host modulation in an ant–cestode system

**DOI:** 10.1186/s12864-026-12581-6

**Published:** 2026-01-31

**Authors:** Tom Sistermans, Romain Libbrecht, Susanne Foitzik

**Affiliations:** 1 Institute of Organismic and Molecular Evolution, Johannes Gutenberg University Mainz, Hanns-Dieter-Hüsch-Weg 15, D-55128 Mainz, Germany; 2 Insect Biology Research Institute, UMR 7261, CNRS, University of Tours, Tours, France

**Keywords:** Interactome, WGCNA, Gene networks, Parasite manipulation, Social insects

## Abstract

How parasites interact with their hosts at the molecular level is a central question in parasitology, yet identifying host pathways directly targeted by parasites is challenging because infections often have broad effects on host physiology. This difficulty is particularly pronounced in non-model systems, such as the interaction between the parasitic tapeworm *Anomotaenia brevis* and its intermediate host, the ant *Temnothorax nylanderi*, in which infection induces strong phenotypic changes. Here, we integrated host and parasite transcriptomes through a combined weighted gene co-expression network analysis (WGCNA) to identify candidate genes and gene networks involved in this interaction. We detected strong negative correlations between parasite and host gene expression, whereas within-species associations were largely positive. Candidate parasite genes were associated with host molecular pathways relevant to infection and host phenotype. The gene networks and expression correlations identified were consistent with those described in model parasite–host systems, supporting the robustness of our approach. Besides, our analysis provided initial functional insights into previously unannotated parasite proteins that may act as effectors of host manipulation. Expression of these parasite genes was correlated with host genes involved in oxidative stress resistance, metabolism, muscle function, immunity, and cuticular sclerotization. These associations suggest that the parasite may modulate multiple host pathways to facilitate infection and transmission. Overall, our findings advance our understanding of molecular mechanisms underlying parasite interference and highlight the value of integrating host and parasite transcriptomic data. More generally, our combined WGCNA framework provides a useful tool for uncovering transcriptional interactions in complex host–parasite systems.

**Supplementary Information:**

The online version contains supplementary material available at 10.1186/s12864-026-12581-6.

## Introduction

Parasite infections can profoundly alter the phenotypes of their hosts. These changes can be multifaceted and include behavioural shifts [1, 2], physiological alterations [3, 4] and immunological changes [5]. While these alterations are often the result of active manipulation by parasites to enhance their fitness [6], alternative mechanisms may contribute to phenotypic changes of hosts, including selectively neutral or detrimental side-effects of parasite manipulation, interference by other organisms, or host defenses [6–8]. Identifying the molecular mechanisms and signalling pathways underlying these phenotypic changes in the host is often challenging, especially in non-model organisms.

Investigating the molecular processes that mediate infection-related phenotypic changes in hosts is even more difficult when multiple phenotypes are affected. For example, when workers of the ant *Temnothorax nylanderi* are infected by the cestode *Anomotaenia brevis*, they show diverse phenotypic changes such as sluggish behaviour [9], muscle dystrophy [10], significant lifespan extension [11] and reduced cuticle sclerotization and melanization [12]. *A. brevis* infection can affect up to 10% of the transcriptome of the host workers [13], therefore the high diversity of infection-induced changes make the search for the parasite-targeted signalling pathways difficult. Despite the complexity of this system, identifying the pathways underlying these phenotypic alterations can provide key insights into the genotype–phenotype map and advance our understanding of both symbiotic interactions and organismal biology in non-model systems.

In order to identify candidate genes and signalling pathways of the parasite that elicit changes in the activity of host genes and pathways, several forms of bioinformatic predictions of host-parasite protein interactions (HPPI) are typically employed [14–16]. Firstly, there are homology-based predictions, which expect that homologues retain conserved functions, secondly, there are domain- and motif-based HPPI predictions, which assume that they interact with functional domains of proteins that bind them. Although they have been proven to be useful in model organisms, for non-model organisms these methods possess several limitations. Homology-based predictions are limited by their ability to identify novel gene functions that evolved in the parasite through adaptation to its novel lifestyle and when protein functions have diverged [17, 18]. This becomes especially difficult when considering that parasite genomes are often shaped by evolutionary arms races in which the neofunctionalization of genes is common [19]. Meanwhile, domain- and motif-based HPPI predictions are computationally intensive and difficult to apply to non-model species with poorly annotated genomes, which constitute the majority of host–parasite associations. Consequently, identifying parasite genes that target host molecular pathways in these systems remains challenging.

A less computationally intensive method that only requires parasite and host transcriptomes is the usage of Weighted Gene Co-expression Network Analysis (WGCNA; [20]). This method constructs gene networks, or modules, based on correlated gene expression, with highly interactive hub genes linking network components. Such hub genes have been identified as key regulators of the human immune response to tuberculosis [21] and to the pathogenic fungus *Candida albicans* [22]. In non-model organisms, WGCNAs helped to identify gene modules involved in *Ophiocordyceps kimflemingiae – Camponotus castaneus* interactions [23], by correlating host and parasite module eigengene values and inferring biological functions from significant co-correlations.

Although these approaches are informative, identifying parasite genes that directly interact with the host transcriptome remains challenging because correlations based on module eigengenes represent only indirect associations between host and parasite expression patterns. We therefore adopted a more direct strategy by analyzing individual host and parasite transcriptomes jointly, rather than correlating host and parasite gene modules derived from separate, species-specific analyses. Our approach was applied to a published transcriptome dataset involving the parasitic tapeworm *Anomotaenia brevis* and its intermediate host, the ant *Temnothorax nylanderi*, in which strong parasite-induced effects on host gene expression, physiology, behaviour, and lifespan have been documented [10–12, 24, 25]. We integrated expression data from haemolymph-residing cysticercoid larvae of the cestode and the fat body of individual host workers to construct a joint co-expression network comprising genes from both species. We hypothesized that, if the parasite actively manipulates host transcription, parasite genes involved in manipulation would show strong correlations with host genes underlying phenotypic changes, resulting in tighter parasite–host links in contrast to within-species gene associations. Using this framework, we identified parasite genes linked to host genes and signalling pathways, providing initial functional insights into previously uncharacterized genes in both species. From the host perspective, we detected gene networks closely integrated with the parasite transcriptome and, through gene ontology analyses, identified host biological processes affected by parasitism in the interaction between *T. nylanderi* and *A. brevis*. Furthermore, leveraging a recent publication of the *A. brevis* secretome [26], we evaluated the potential roles of previously unannotated secreted proteins through their expression links to host genes. This straightforward, integrative approach is applicable to other non-model systems and can help elucidate the molecular mechanisms underlying parasite–host interactions.

## Materials and methods

### Combined WGCNA analysis

We obtained our transcriptome data from Sistermans et al. (2025; methodological details see supplement [13]). Raw reads are published under the SRA database of NCBI (PRJNA1246159), and the gene count matrices, GO terms and KEGG terms on Dryad (DOI: 10.5061/dryad.8cz8w9h3b). We targeted the host’s fat body as in insects, this physiologically active organ is responsible for synthesizing and processing proteins essential for immunity, fecundity, and longevity [27]. Gene counts were paired per sample (table S1); as both originated from the same worker ant. This process yielded a joint gene count matrix for 15 samples, encompassing transcripts from both cestode and ant genes (Table S1). From this combined matrix, we filtered out genes with fewer than ten counts in at least five samples and verified the data for missing entries using the WGCNA package (version 1.72-2) [20] in R (version 4.3.2). To construct an unsigned co-expression network, we set the soft-thresholding power to 8. After testing various module sizes, we established a minimum module size of 100 and confirmed this using a TOM plot. Modules with a dissimilarity threshold of 0.2 were merged, and the result was validated with an additional TOM plot (Fig. S1). We identified hub genes by selecting the genes with the top 10% highest connectivity within their modules. We used a chi-square test per module to check whether there were more cestode hub genes than would be expected by chance. For this, we used the proportion of cestode genes per module as the expected variable, the proportion of cestode hub genes as the observed variable and the number of hub genes as the sample size. We corrected the p-values for multiple testing using Benjamini-Hochberg adjustment. We converted module-specific topological overlap matrices into Cytoscape objects (Cytoscape version 3.26.0) [28] to visualize weighted gene–gene correlations and construct parasite–host co-expression networks. These networks were used to test whether genes preferentially correlated with genes from the other species, relative to expectations under random association based on the proportion of ant and cestode genes within each module (e.g., a 50:50 ratio predicts equal proportions of correlations with genes from both species). Although gene expression within the same species is likely to be more strongly statistically linked, we tested against the more conservative null hypothesis of random correlations. We calculated the proportion of genes that are correlation partners of cestode genes for both ant and cestode genes and took their respective averages in each module, we then tested deviations from the null hypothesis using a chi-square test, with the number of ant or cestode genes in each module as the sample size. P-values were adjusted for multiple testing using the Benjamini-Hochberg method resulting in conservative estimates.

### Strength and directionality of within and between species expression links

We assessed the directionality and strength of host–host, parasite–parasite, and host–parasite gene–gene correlations. Because WGCNA does not provide traditional Pearson *r* values, we performed a separate analysis by calculating Pearson correlations for all gene pairs in the combined expression matrix using R. We then excluded correlations between genes from different modules and all non-significant correlations. Owing to the size of the resulting dataset, we randomly subsampled 1,000 correlations, which were analysed using a linear model followed by ANOVA and Tukey HSD tests. Correlation coefficients were visualized using density plots and boxplots.

### Links to genes differentially expressed between infected and uninfected workers

With our approach, we were unable to include uninfected samples, although they were present in the original dataset [13]. This limitation stems from treating host and parasite transcriptomes as a single dataset, as including uninfected controls would introduce zero counts for all parasite genes, biasing module construction and likely forcing cestode genes into separate modules. To assess whether host genes whose expression changes following infection are associated with cestode transcriptional activity, we tested whether these genes were more likely to be linked to cestode genes or to function as hub genes within modules, as expected if they are targeted by parasite genes in the combined WGCNA analysis. We identified differentially expressed genes (DEGs) between infected and uninfected ants using DESeq2 (v1.50.2) [29]. We applied a likelihood ratio test (LRT), comparing 15 infected and 15 uninfected samples, with parasite infection as the dependent factor and colony identity as the reduced factor. Although additional metadata were available (cestode number, colony size, and colony infection rate), these variables were not included because cestode number is nested within infection status, and colony size and infection rate are fully nested within colony identity. We tested whether DEGs were overrepresented among hub genes using Fisher’s exact tests. We further examined whether DEGs were more strongly correlated with cestode or ant genes than expected by chance. To do so, we calculated confidence intervals for the proportion of cestode genes per module. Given the number of tests, Z-scores were Bonferroni-corrected by dividing the significance threshold by the number of comparisons. For each DEG, we then assessed whether the proportion of correlated cestode genes fell outside the confidence interval. Values below the interval indicate stronger correlations with ant genes, whereas values above indicate stronger correlations with cestode genes. Additional analyses, including DEG module membership and associated KEGG pathways, are provided in the Supplementary Material.

### Links to effector proteins secreted by the parasite into the host

Ninety-eight proteins were previously shown to be consistently secreted by the cestode into the ant haemolymph and may therefore be regarded as effector proteins [26]. We selected the 15 most abundant proteins in the cestode secretome and all annotated genes among the 50 most abundant secreted proteins. These genes were used as queries for a DIAMOND BLAST search against a custom database created from our genome coding sequences (CDS), which were translated using TransDecoder v5.5.0 [30]. For each query, the most frequently expressed gene among the BLAST hits was selected to identify the corresponding gene in the proteome [26]. Using these identified genes, we determined the modules in which they were located and evaluated whether these modules contained ant or cestode gene clusters that were more strongly associated with the other species. Confidence intervals were calculated for these modules, and we tested whether these genes were more likely to correlate with genes from the other species by assessing whether their correlations fell outside the 95% confidence interval. We then identified the ant genes correlated with these cestode genes and conducted GO and KEGG enrichment analyses to infer their potential functions, using annotation files derived from Sistermans et al. (2025) [13].

## Results

### Combined WGCNA analysis

**Fig. 1:**
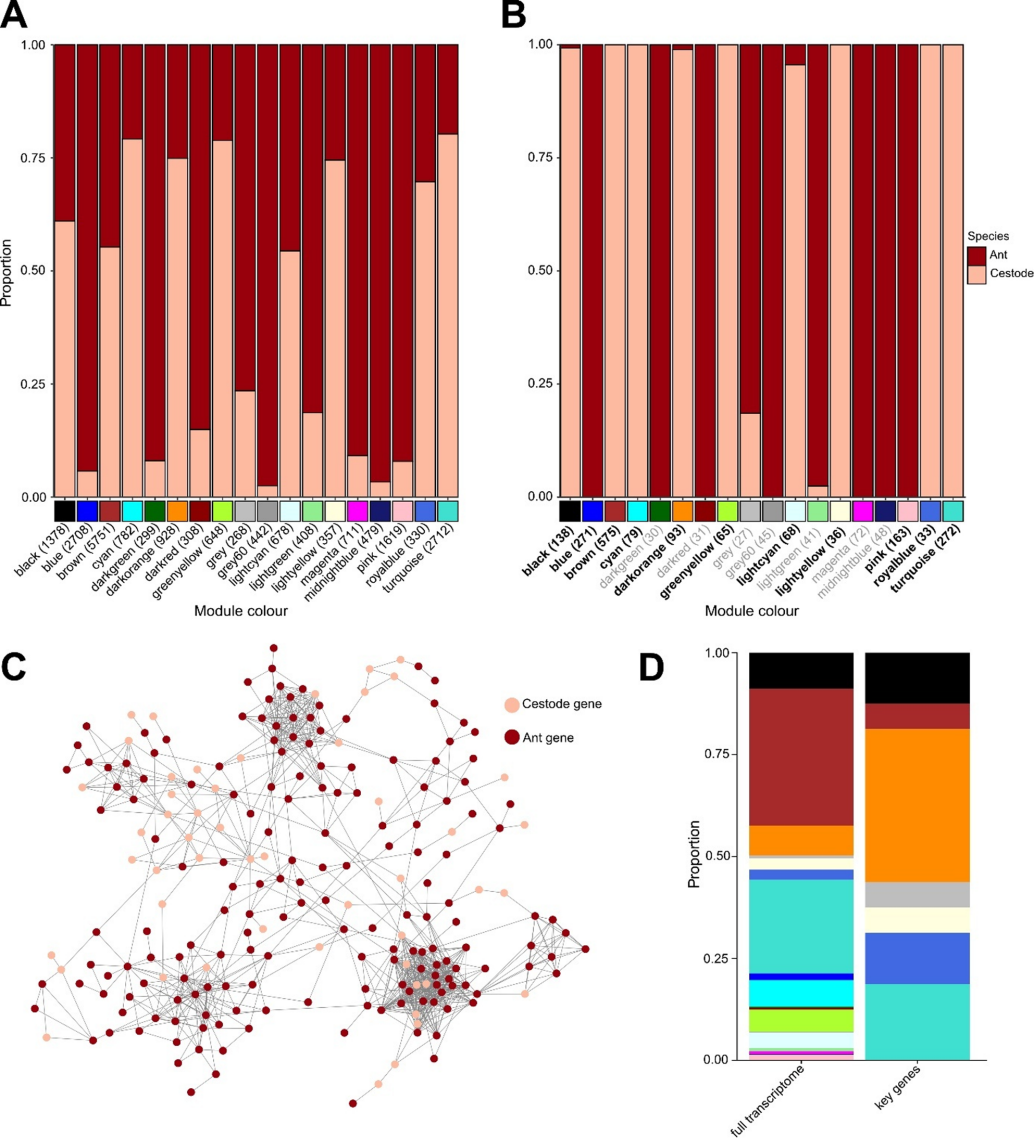
Results of the interactome analysis.** A **WGCNA modules and gene composition. Distribution of genes across modules, separated by species. Cestode genes are shown in pink and ant genes in brown. Module size is indicated on the x-axis alongside module names. **B **Hub gene composition. Species composition of hub genes in each module, using the same colour scheme. The number of hub genes is indicated next to each module name; module names in bold indicate significant overrepresentation of either parasite or host genes. Modules composed entirely of hub genes from one species do not necessarily deviate significantly from overall module composition. **C **Gene correlations within the grey module. Network representation of all significant gene–gene correlations. Nodes represent individual genes and edges indicate significant correlations. Cestode genes are shown in pink and ant genes in brown; spatial distance between nodes does not reflect correlation strength. **D **Module membership of cestode genes. The left panel shows module membership for all cestode genes, while the right panel highlights the module membership of the 16 most frequently detected cestode proteins in the secretome [26]

For our analysis, we combined gene count matrices from hosts and their corresponding parasites and analysed gene–gene correlations using WGCNA. From the combined host and parasite dataset, we identified 18 WGCNA modules comprising a total of 20,806 genes, including 9,472 cestode genes and 11,334 ant genes. Module sizes ranged from 261 to 5,751 genes. (Fig. 1a). These modules encompassed genes from both species, with the proportion of cestode genes per module varying between 0.02 and 0.8 (Fig. 1a). When identifying hub genes, genes with the top 10% connectivity, we demonstrated that in most modules, the proportion of hub genes significantly differed from the overall proportion of genes from each species within the respective module (Fig. 1b; p-values Table 1, column 5). Indeed, in seven modules all hub genes originated from the host, with two modules (blue and pink) containing significantly more host hub genes than expected by chance, whereas in six modules all hub genes were of cestode origin. Overall, 11 of the 18 modules exhibited a significantly higher proportion of cestode hub genes than ant hub genes (Table 1). In these modules, the proportion of cestode genes ranged from 0.55 to 0.80.

**Table 1:** Statistical summary of correlome modules. Black values indicate stronger correlations with the other species, grey values stronger within-species correlations. Bold p-values denote statistical significance or significantly stronger interspecific correlations

Module ID	Proportion of cestode genes	*P*-adjusted ant genes correlating differently from proportion of ant-cestode genes	*P*-adjusted cestode genes correlating differently from proportion of ant-cestode genes	*P*-adjusted cestode genes hub representation compared to proportion
Black	0.61	**< 0.0001**	0.0001	**< 0.0001**
Blue	0.06	0.002	1	**< 0.002**
Brown	0.55	**< 0.0001**	< 0.0001	**< 0.0001**
Cyan	0.79	**0.01**	< 0.0001	**0.0007**
Darkgreen	0.08	0.12	1	1
Darkorange	0.75	**0.0002**	< 0.0001	**< 0.0001**
Darkred	0.15	0.19	1	0.40
Greenyellow	0.79	0.07	0.001	**0.003**
Grey	0.23	0.31	1	1
Grey60	0.02	0.31	1	1
Lightcyan	0.54	**0.0002**	0.0003	**< 0.0001**
Lightgreen	0.19	0.0003	1	0.26
Lightyellow	0.75	0.11	0.001	**0.03**
Magenta	0.09	0.0008	1	0.19
Midnightblue	0.03	0.31	1	1
Pink	0.08	< 0.0001	1	**0.01**
Royalblue	0.70	0.31	0.0008	**0.02**
Turquoise	0.80	**< 0.0001**	< 0.0001	**< 0.0001**

Visualization of the networks using Cytoscape [28] revealed interspecific gene correlations (Fig. 1c). This was further supported by the correlation analysis, in which we tested whether genes from one species were more likely to correlate with genes from the other species than expected under the null hypothesis, defined as the proportion of ant and cestode genes within each module. Thus, if the proportion of cestode genes in a module was 0.5, we tested whether cestode gene correlation partners consisted of 50% cestode genes and 50% ant genes, or whether this distribution differed significantly in either direction. After p-value adjustment, we identified six modules in which ant genes were significantly more likely to correlate with cestode genes than expected by chance (Table 1). In contrast, we found no modules in which cestode genes showed higher-than-expected correlations with ant genes.

### Strength and directionality of within and between species expression links

**Fig. 2:**
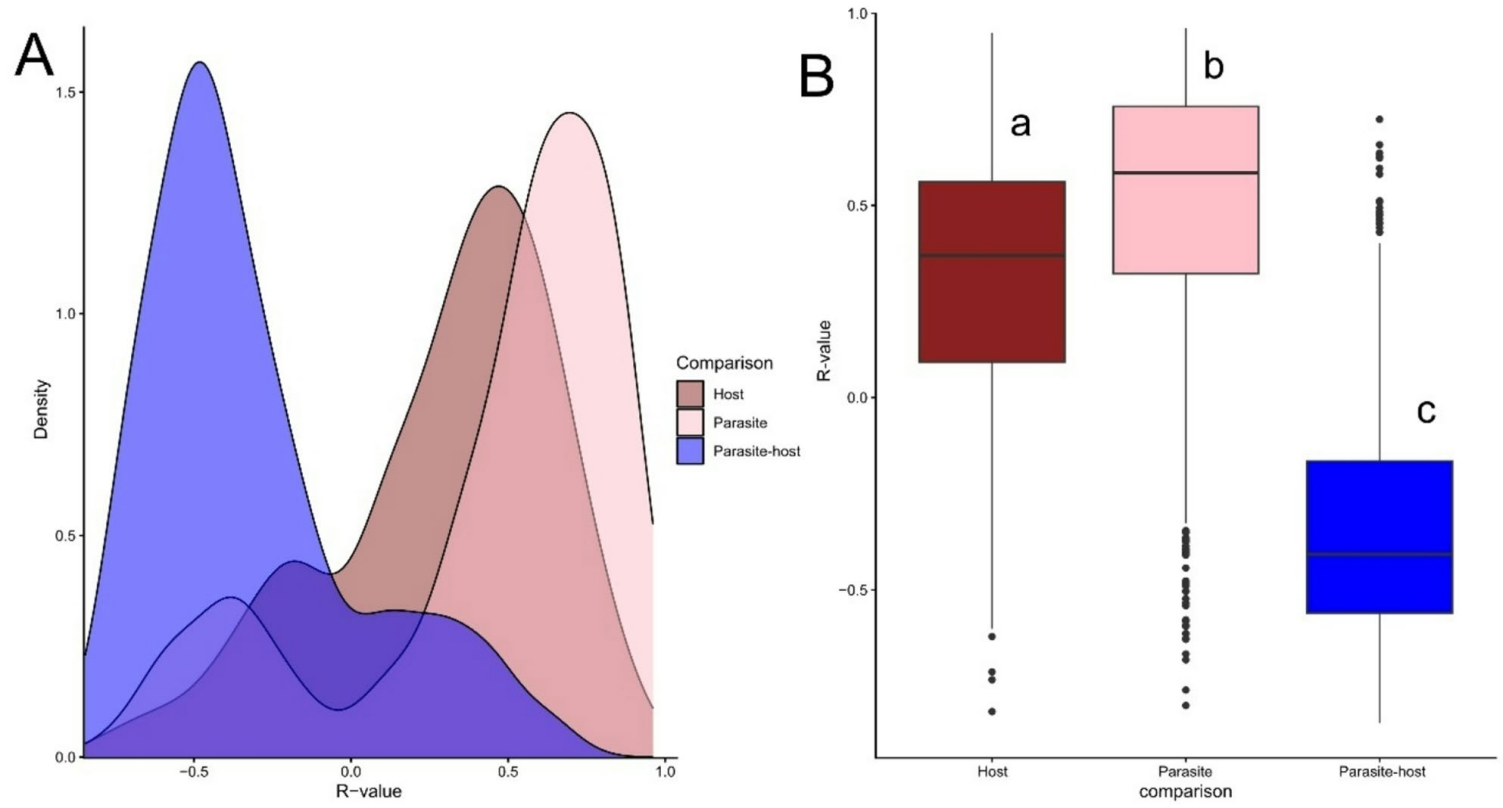
The strength and directionality of parasite-host and within species links of gene expression based on a random sample of 1000 genes. **A** Density plot where color signifies which correlation, pink being parasite-parasite, brown host-host and blue parasite-host. **B** A boxplot where each box signifies the correlation, the colors are the same as in Fig. 2A. The letters indicate that all three groups differ from each other significantly

We assessed the strength and direction of gene expression correlations within and between species. Parasite–host correlations were predominantly negative, whereas parasite–parasite and host–host correlations were mostly positive (Fig. 2a). All three correlation groups differed significantly (Tukey’s HSD: all pairwise comparisons, *P* < 0.0001; Fig. 2b), with the strongest effect observed for parasite–host correlations. We next compared correlation strength using the absolute values of *r*, independent of sign. Parasite–parasite correlations were significantly weaker than both parasite–host and host–host correlations (Tukey’s HSD: *P* < 0.0001 for both comparisons). In contrast, host–host and parasite–host correlations did not differ in strength (*P* = 0.766).

### Links of genes differentially expressed between infected and uninfected workers

We identified a total of 683 DEGs associated with parasite infection in ant workers, of which 315 were overexpressed and 368 were downregulated in infected ants. There was a non-significant trend that DEGs were more likely to be hub genes (Fisher’s exact test; *P* = 0.088). Testing whether DEGs preferentially correlated with cestode genes yielded strong support for this pattern (Table S4): about half of all DEGs between infected and uninfected workers, namely 320 (47%), were more strongly associated with cestode genes, 52 more strongly associated with ant genes, while for 309 DEGs associations did not deviate from the null hypothesis.

### Links of effector proteins secreted by the parasite into the host

**Fig. 3:**
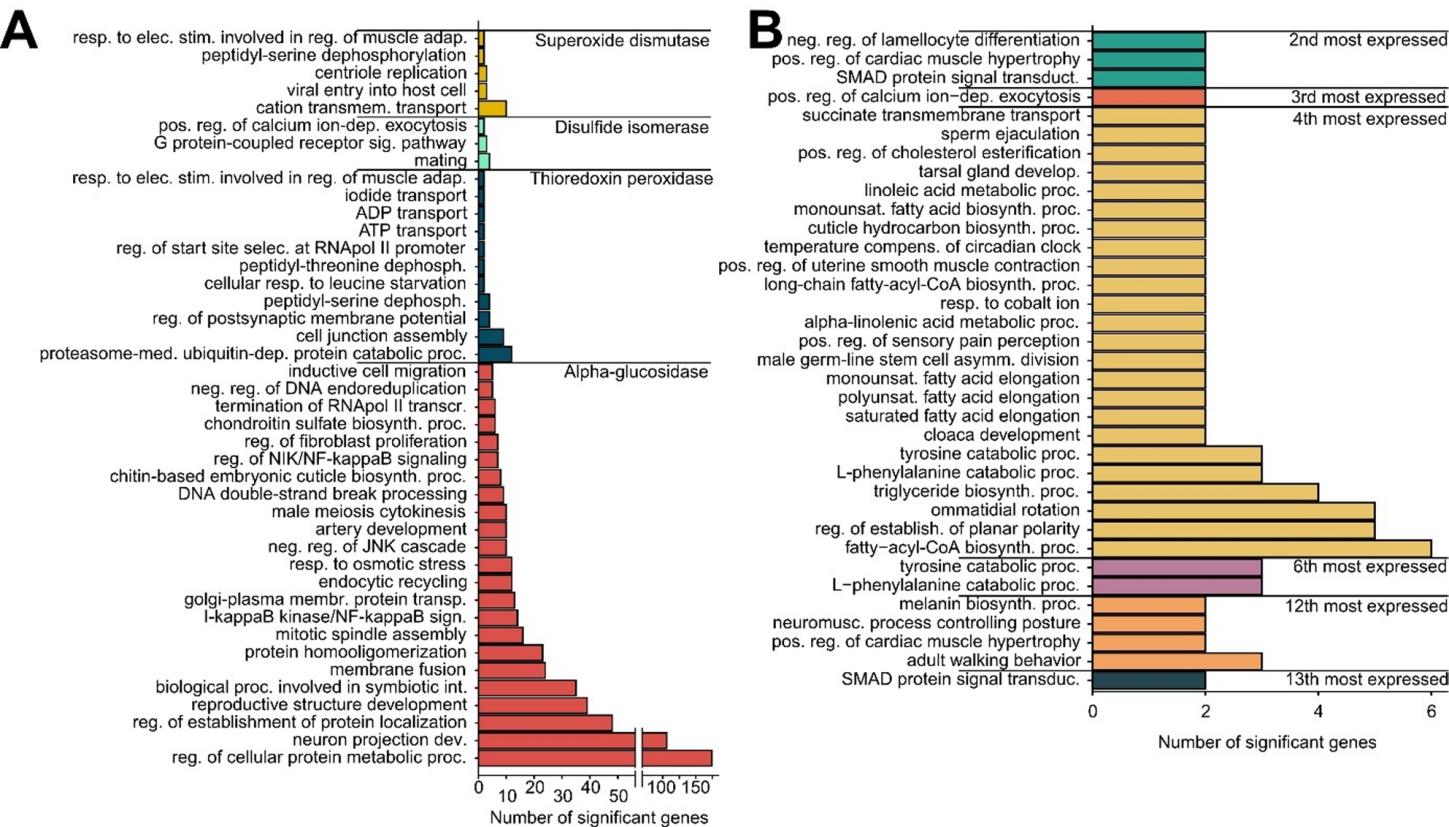
Gene Ontology enrichment of ant correlation partners.** A **Annotated cestode genes. GO enrichment of ant correlation partners for annotated, commonly secreted cestode genes detected in the ant haemolymph. Different colours indicate correlation partners associated with individual cestode genes. **B **Unannotated cestode genes. GO enrichment of ant correlation partners for unannotated, commonly secreted cestode genes. Colours represent correlation partners of individual genes, with the degree of commonality indicated on the right side of the bars

Cysticercoid larvae of *Anomotaenia brevis*, which reside in the haemolymph and are loosely attached to the host gut, have been shown to secrete proteins into the host haemolymph [26]. We identified four annotated and twelve unannotated transcripts of the most abundant cestode proteins in our cestode transcriptomes (Fig. 1d; Table 2) and identified the correlation partners of these genes separately. The four annotated genes include *thioredoxin peroxidase* and *superoxide dismutase* (black module), *lysosomal alpha-glucosidase* (brown module), and a putative *protein disulfide isomerase ER 60* (dark orange module). These genes exhibited correlation patterns that differed from what their proportional representation in the modules would predict (Table 2). Specifically, *superoxide dismutase* correlated predominantly with ant genes, whereas the other genes showed stronger links to cestode genes than expected (Table 2). Notably, two of these genes, *protein disulfide isomerase* and *alpha-glucosidase*, were identified as hub genes. Gene Ontology (GO) enrichment analysis of the ant correlation partners of these genes (Fig. 3a) revealed significant GO terms associated with their functions.

**Table 2:** Expression links of the most abundant cestode secretome genes. Genes are ranked by abundance in the ant haemolymph. For each gene, we report module assignment, gene name [26], the proportion of correlated cestode genes, the proportion of cestode genes in the module, standard error, confidence interval bounds, and whether expression is primarily linked to ant genes, cestode genes, or shows no significant bias

Gene rank	Module color	Gene name		Prop. cestode genes correlated with		Prop. of cestode genes in module		SE		Lower bound		Upper bound		Significant	
1	Turquoise	TRINITY_DN231_c0_g2_i2_1	0.988		0.869		0.007		0.854		0.883		Cestode		
2	Dark orange	TRINITY_DN141_c0_g1_i5_2	0.855		0.889		0.012		0.865		0.912		Ant		
3	Dark orange	TRINITY_DN66_c1_g1_i1_3	0.914		0.889		0.012		0.865		0.912		Cestode		
4	Turquoise	TRINITY_DN943_c0_g1_i2_4	0.824		0.869		0.007		0.854		0.883		Ant		
5	Grey	TRINITY_DN143_c0_g1_i9_5	0.4		0.372		0.065		0.244		0.5		N.S.		
6	Turquoise	TRINITY_DN199_c0_g2_i1_6	0.945		0.869		0.007		0.854		0.883		Cestode		
8	Light yellow	TRINITY_DN211_c0_g1_i5_8	0.857		0.884		0.02		0.845		0.922		N.S.		
9	Black	TRINITY_DN4073_c0_g2_i2_9_thioredoxin_peroxidase	0.652		0.714		0.016		0.684		0.745		Ant		
10	Royal blue	TRINITY_DN53_c1_g1_i7_10	0.934		0.858		0.023		0.813		0.903		Cestode		
12	Dark orange	TRINITY_DN25_c0_g2_i4_12	0.843		0.889		0.012		0.865		0.912		Ant		
13	Dark orange	TRINITY_DN81_c0_g1_i7_13	0.882		0.889		0.012		0.865		0.912		N.S.		
14	Royal blue	TRINITY_DN117_c0_g1_i9_14	0.827		0.858		0.023		0.813		0.903		N.S.		
15	Dark orange	TRINITY_DN22_c0_g1_i4_15	0.94		0.889		0.012		0.865		0.912		Cestode		
22	Black	TRINITY_DN897_c0_g1_i2_22_superoxide_dismutase	0.778		0.714		0.016		0.684		0.745		Cestode		
31	Dark orange	TRINITY_DN3503_c0_g2_i2_31_putative_protein_disulfide_isomerase_ER_60	0.828		0.889		0.012		0.865		0.912		Ant		
42	Brown	TRINITY_DN16034_c0_g1_i1_42_lysosomal_alpha_glucosidase	0.581		0.665		0.008		0.648		0.681		Ant		

For the ant genes that were statistically linked to the cestode *superoxide dismutase*, two enriched GO terms were identified: *cation transmembrane transport* (GO:0098655; 10 genes; significant GO terms of annotated genes in table S2) and *response to electrical stimulus involved in regulation of muscle adaptation* (GO:0014878; 2 genes). Transmembrane cation transport and muscle contraction can both increase reactive oxygen species (ROS) levels [31, 32], potentially explaining the association between this cestode gene and ant genes with these functions. This aligns with the established role of *superoxide dismutase* in regulating oxidative stress, as demonstrated in *Caenorhabditis elegans* [33], and its expression has also been associated with increased lifespan in queens of several social insects [34–36]. In parasitic helminths, *superoxide dismutase* can play a role in defense against ROS originating from the host [37]. In the same module (black), we found another antioxidant, *thioredoxin peroxidase* [38] involved in the regulation of oxidative stress associated with normal aerobic metabolism [39], which with the enriched GO terms of the correlating host genes, including several metabolic processes such as *proteasome-mediated ubiquitin-dependent protein catabolism* (GO:0043161; table S2), *peptidyl-threonine dephosphorylation* (GO:0035970), and *regulation of transcriptional start site selection at the RNA polymerase II promoter* (GO:0001178). Additionally, pathways involved in the ant’s defense against oxidative stress were identified, including *ADP transport* (GO:0015866), which plays a protective role in mitigating oxidative stress [40]. The concordance between these results and those for *superoxide dismutase* likely reflects the strong overlap in correlated host genes: 190 of 191 *superoxide dismutase* partners were also among the 428 *thioredoxin peroxidase* partners.

The third gene, the putative *protein disulfide isomerase ER 60*, belongs to a gene family known to influence parasite virulence [41] and its downregulation in parasitic nematodes can increase parasite mortality in host plants [42]. The most common GO term, represented by four genes, is *mating* (GO:0007618; table S2), a process in which disulfide isomerases play a direct role; in vertebrates, they act as chaperones for ADAM3, whose misfolding leads to male infertility [43]. In parasites, this gene can reduce host fertility by increasing the production of secretory proteins, which leads to a reduction in mating factor binding [44]. Disulfide isomerases have further been found to act as chaperones for other proteins as well [45, 46]. This chaperone function could explain the GO term *positive regulation of calcium ion-dependent exocytosis* involving two genes, as exocytosis is a key component of the secretory pathway [47]. This may be relevant to the study system, as infected hosts are more likely to become fertile following queen removal [48]. The final annotated gene with identified correlation partners is *lysosomal alpha-glucosidase* from the brown module, which supports basal metabolism by degrading glycogen and releasing stored glucose [49]. In cestodes, the expression of another glucosidase, beta-glucosidase, has been shown to correlate negatively with the expression of a host beta-glucosidase [50]. We examined whether an ant host alpha-glucosidase was among the correlation partners of the cestode *lysosomal alpha-glucosidase* and identified both its presence and a significant expression correlation using a linear model (*p* = 0.03, *r* = 0.113; Fig. S2). GO term enrichment analysis of the correlation partners revealed *regulation of cellular protein metabolic process* (GO:0032268; Table S2), encompassing 174 genes, a pathway closely linked to glucose metabolism and glycolysis [51]. We uncovered further connection to glucose metabolism with the term *response to osmotic stress* (GO:0006970; 12 genes), which can be linked to altered glucose concentrations [52]. Alpha-glucosidases can also influence neuromuscular functions and a deficiency can cause motor and respiratory disorders, known as Pompe disease in vertebrates [53]. It is therefore unsurprising that the most common GO term is *neuron projection development* (GO:0031175) with 106 genes. Pompe disease decreases glycogen concentration in projection neurons [54]. We also identified a cuticle-related GO term, *chitin-based embryonic cuticle biosynthetic process* (GO:0008362), which is relevant because chitin-containing compounds inhibit *alpha-glucosidase* [55], and ants infected during the larval stage exhibit reduced cuticle sclerotization and melanization during pupation.

We also obtained GO terms for the host correlation partners of six commonly secreted cestode proteins that could not be annotated; for the remaining proteins, no significant GO terms were detected among their correlation partners [26]. Four of these genes were located in the dark orange module, representing the second, third, twelfth, and thirteenth most abundant parasitic proteins in the ant haemolymph, while two were in the turquoise module, corresponding to the fourth and sixth most abundant proteins (Fig. 3b). The second most abundant gene had 97 correlation partners and was associated with three significant GO terms, all based on two genes (Fig. 3b). One of these terms is *SMAD protein signal transduction* (GO:0060395; overview of significant GO terms see table S3), a signalling pathway involved in muscle hypertrophy and the negative regulation of muscle growth [56]. This term co-occurs with *positive regulation of cardiac muscle hypertrophy* (GO:0010613). Further analysis of the dark orange module showed that these cestode genes strongly correlate with host genes involved in the SMAD signalling pathway (*SMAD protein signal transduction*, GO:0060395; 13th most enriched term; Table S3) and other muscle-related processes, including *neuromuscular process controlling posture* (GO:0050884), *positive regulation of cardiac muscle hypertrophy* (GO:0010613), and *adult walking behaviour* (GO:0007628; 12th most enriched term; Table S3). As in infected ants show lower activity [9] and muscular dystrophy–like symptoms [10], these associations may provide insight into the molecular mechanisms underlying the observed muscle dysfunction. We also identified two immune-related functions among these correlating host genes, including the *melanin biosynthetic process* (GO:0042438), which plays a critical role in the encapsulation of foreign substances [57] and *negative regulation of lamellocyte differentiation* (GO:0035204), lamellocytes being key insect immune cells [58]. We detected similar GO terms among the correlation partners of the fourth and sixth most abundant cestode proteins in the ant haemolymph located in the turquoise module. These proteins correlate with host genes enriched for *tyrosine catabolic process* (GO:0006572) and *L-phenylalanine catabolic process* (GO:0006559). The tyrosine pathway is involved in sclerotization and melanization of the insect cuticle [59], another GO term of which can be found in *cuticle hydrocarbon biosynthetic process* (GO:0006723) correlating with the fourth most expressed gene. Moreover, phenylalanine catabolism plays a crucial role in the encapsulation of malaria parasites in mosquitoes. When phenylalanine catabolism is disrupted, for example, through the silencing of phenylalanine hydroxylase, this encapsulation process is impaired [60] and mosquitoes became unable to encapsulate malaria parasites [61].

The expression of the fourth most abundant protein is associated with host genes involved in lipogenesis, for which we identified nine enriched GO terms (GO:0010873, GO:0043651, GO:1903966, GO:0035338, GO:0036109, GO:0034625, GO:0034626, GO:0019367 and GO:0019367; table S3). Considering that the fat body is the most important immune organ in insects [62], fat metabolism and immunity are likely interconnected. While our GO analysis did not identify specific immune functions beyond *L-phenylalanine catabolic process* (GO:0006559) and the *response to cobalt ion* (GO:0032025) involved in detoxification, the involvement of genes in lipogenesis may provide insights into how the immune system of infected ants is altered. For instance, in *Drosophila* lipogenesis is suppressed by the Toll signalling pathway during periods of immune stress [63], and lipids themselves can play a role in the immune responses against fungal parasites [64].

## Discussion

By applying Weighted Gene Co-expression Network Analysis (WGCNA) to a combined dataset of host and parasite transcriptomes, we examined how host and parasite transcriptional activities are linked. Parasite–host gene expression correlations were mainly tight and negative, whereas correlations within each species were mostly positive. About half of the host genes that were differentially expressed between infected and uninfected workers showed stronger links to the expression of cestode genes, pointing to a direct influence of the parasite on host transcription. Furthermore, in multiple modules parasite gene expression was connected to a larger number of genes than host gene expression. This is evident by the fact that in six modules, host genes were more likely to correlate with parasite genes than with other host genes, while cestode genes in these modules mainly correlated with other cestode genes. This pattern is consistent with weaker connectivity among host genes compared with the higher connectivity observed among cestode genes.

This effect is particularly evident in the hub genes of these modules. In modules where ant gene correlation partners were significantly more represented by cestode genes than the proportion of cestode genes in the module, the representation of hub genes was overwhelmingly dominated by cestode genes. One plausible explanation is that gene expression in *A. brevis* is tightly coupled to that of *T. nylanderi*, such that host expression changes directly affect the parasite. In contrast, host gene expression is influenced by multiple social, biotic, and abiotic factors, making it less strongly shaped by the parasite alone.

For instance, during a pathogenic infection, the host activates immune responses that primarily alter gene expression in the fat body, with limited effects on the rest of the transcriptome. In contrast, the same immune challenge may require the cestode to activate its own immune defences to prevent hyperparasitism [65, 66], modulate the host immune response [67], and adapt to a more hostile environment generated by immune activation [68]. This would result in co-expression among parasite stress, immune, and host–parasite communication pathways, while changes in the host remain largely restricted to immune genes. If the parasite directly influences host gene expression, it may benefit from limiting host physiological disruption by adjusting its own transcription more strongly to stabilize host metabolism.

We also observed a strong bias toward negative correlations between parasite and host gene expression. Similar patterns have been reported previously for individual genes, such as parasite and host α-glucosidase, as noted above [50]. Negative associations have also been found at the level of gene clusters, for example in a study of human and malaria transcriptomes, where parasite and host module eigengenes showed clear negative correlations [69]. However, the overall dominance of negative parasite–host correlations observed here is novel and may provide insight into the evolution of host–parasite interactions. Our results suggest that parasites may primarily alter host phenotype by inhibiting host gene expression. This interpretation is consistent with well-established evidence that parasites often suppress host immune responses [70, 71]. The tight coupling between host and parasite gene expression was also evident in our analysis of genes that change expression with infection in ant workers. Approximately half of these differentially expressed host genes showed stronger correlations with parasite genes than with host genes in the WGCNA.

Beyond broad host–parasite gene expression patterns, we analyzed correlation partners of genes effector proteins commonly secreted into the ant haemolymph. For annotated genes, their functions closely matched ant biological processes that were either analogous to, or likely influenced by, their parasite functions. Notably, the oxidative stress genes *superoxide dismutase* and *thioredoxin peroxidase* showed strongly linked expression: they clustered in the same module, and *superoxide dismutase* correlated with all but one of the genes correlated with *thioredoxin peroxidase*. This finding aligns with previous studies, which have shown that *superoxide dismutase* and *thioredoxin peroxidase* function within the same oxidative stress pathway in systems without parasite involvement [72] and in parasitic cestodes [39]. The genes they correlate with indicate that their primary role is not necessarily in direct communication with the host but rather in defense against the oxidative stress generated by the host. It has been hypothesized that *thioredoxin peroxidase*, in particular, is secreted by parasites into the host primarily to modulate oxidative stress, which arises naturally in the host through aerobic metabolism [39, 73]. This conclusion is supported by the predominance of metabolic functions among the associated genes and their correlations with other host genes involved in mitigating oxidative stress. Such modulation of oxidative stress may contribute to the markedly extended host lifespan. However, the high secretion levels of these proteins likely did not evolve specifically for this effect, as these genes are among the most highly expressed across many parasite–host systems [74]. Thus, their contribution to lifespan extension could be an incidental by-product rather than an adaptive trait. What becomes evident from the presence of *thioredoxin peroxidase* is that *A. brevis* seems to be able to thrive in its cysticercoid stage in an aerobic environment within the host. This ability is notable, as Platyhelminthes are known to adapt to anaerobic environments at various life stages [75].

For *disulfide isomerase*, we found that its correlation partners are predominantly linked to secretion, consistent with the established functions of disulfide isomerases in parasites and other systems [76, 77]. The last annotated gene, *alpha-glucosidase*, also supports this interpretation. Similar to previous findings with *beta-glucosidase* [50], we observed a negative correlation between parasite *alpha-glucosidase* and host *alpha-glucosidase*. Additionally, its correlation partners are associated with glucose metabolism. *Alpha-glucosidases* are enzymes that catalyze the breakdown of polymeric glucose variants, such as starch or glycogen, into glucose monomers (63). This gene is thus a strong candidate for involvement in the cestode’s nutrient acquisition, as glucose uptake is critical for cestode survival and growth [78]. This activity contrasts with the expected reduction of glycogen levels in infected hosts. Interestingly, metacestode-infected beetles have been reported to exhibit increased glycogen levels (65), potentially as a compensatory response to maintain stable glycogen reserves in the presence of cestode *alpha-glucosidase*. This interplay highlights the intricate metabolic interactions between host and parasite.

Finally, we examined host gene functions associated with unannotated proteins secreted into the ant haemolymph. The four genes in the dark orange module are linked to host genes involved in muscle development and the SMAD signalling pathway, suggesting a potential role in modulating host muscle development and, consequently, host behaviour and morphology [10]. Although *A. brevis* has never been found in the host’s brain, there have been reports on parasites whose infection causes behavioral alterations in social insects while not being present in the brain [79–81]. This could also explain the enrichment of the GO term *walking behaviour*. Together, these observations suggest that these genes are strong candidates for contributing to the host’s sluggish behaviour, and that the high abundance of their protein products in the haemolymph may reflect a need to reach the host brain.

Additionally, the two cestode genes in the turquoise module appear to be involved in immune suppression, potentially with the side effect of inhibiting cuticle sclerotization. This interpretation is supported by the enrichment of genes linked to lipogenesis and immune functions within the module. Given the central role of the fat body in immunity, these patterns are consistent with parasite–host immune interactions, including immune suppression or avoidance, capacities well documented in cestodes [82]. Because this study is limited by the correlative nature of our analyses, we propose that future experiments directly target this host pathway. In adult ants, silencing these genes may restore immune activity against the parasite, potentially resulting in increased expression of serine proteases implicated in cestode immune avoidance [83, 84] and affected by parasite load in our system [24]. Finally, the expression of these genes should be examined in cestode-infected larvae to assess whether they correlate with ant genes involved in cuticle melanization. This approach would allow parasite-mediated immune suppression to be directly linked to a key phenotypic trait of infected ants [12].

## Conclusions

Our analysis reveals strong integration of the parasite transcriptome into that of the host, reflected by high parasite gene connectivity and the presence of cestode genes across all modules. Host–parasite gene expression was predominantly negatively correlated, in contrast to the mainly positive correlations observed within species. Using a novel application of WGCNA, we identified many candidate genes with previously unknown functions, while known candidates were consistent with findings from other studies, supporting the validity of our approach. We therefore conclude that this method is well suited for analysing parasite–host interactomes, particularly in non-model organisms, and could be extended by incorporating multiple host and parasite tissues to identify the tissues where interactions primarily occur.

## Supplementary Information

Supplementary Material 1.

Supplementary Material 2.

## Data Availability

We obtained our data from the published dataset of Sistermans et al. (2025; methodological details see supplement[13]), the raw reads of which can be found on the SRA database of NCBI (PRJNA1246159), and the gene count matrices, GO terms and KEGG terms on Dryad (DOI: 10.5061/dryad.8cz8w9h3b). The script for this analysis and the combined gene count matrix can be found on Mendeley (https://doi.org/10.17632/85w27rg8m9.1).

## Abbreviations

WGCNA: Weighted gene co–expression network analysis
GO: Gene ontology
BLAST: Basic local alignment search tool
KEGG: Kyoto encyclopaedia for genes and genomes
TOM plot: Topological overlap matrix plot
DEG: Differentially expressed genes
