# Supplementary material for: Integrated co-expression analysis of host–parasite transcriptomes reveals mechanisms of host modulation in an ant–cestode system
Source: BMC Genomics. 2026 Jan 31;27:232. doi: 10.1186/s12864-026-12581-6 (PMC12930657; doi:10.1186/s12864-026-12581-6)
Supplement: Supplementary file 1 — Supplementary Material 1. [file 12864_2026_12581_MOESM1_ESM.docx]

**Supplementary materials**


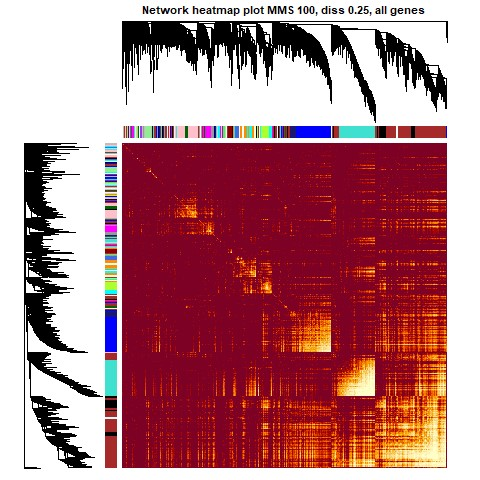


**Figure S1** TOM plot of the merged gene count matrices of both infected ants and their cestodes. The colours representing the constructed modules roughly aligning with the highest correlations in the heatmap.


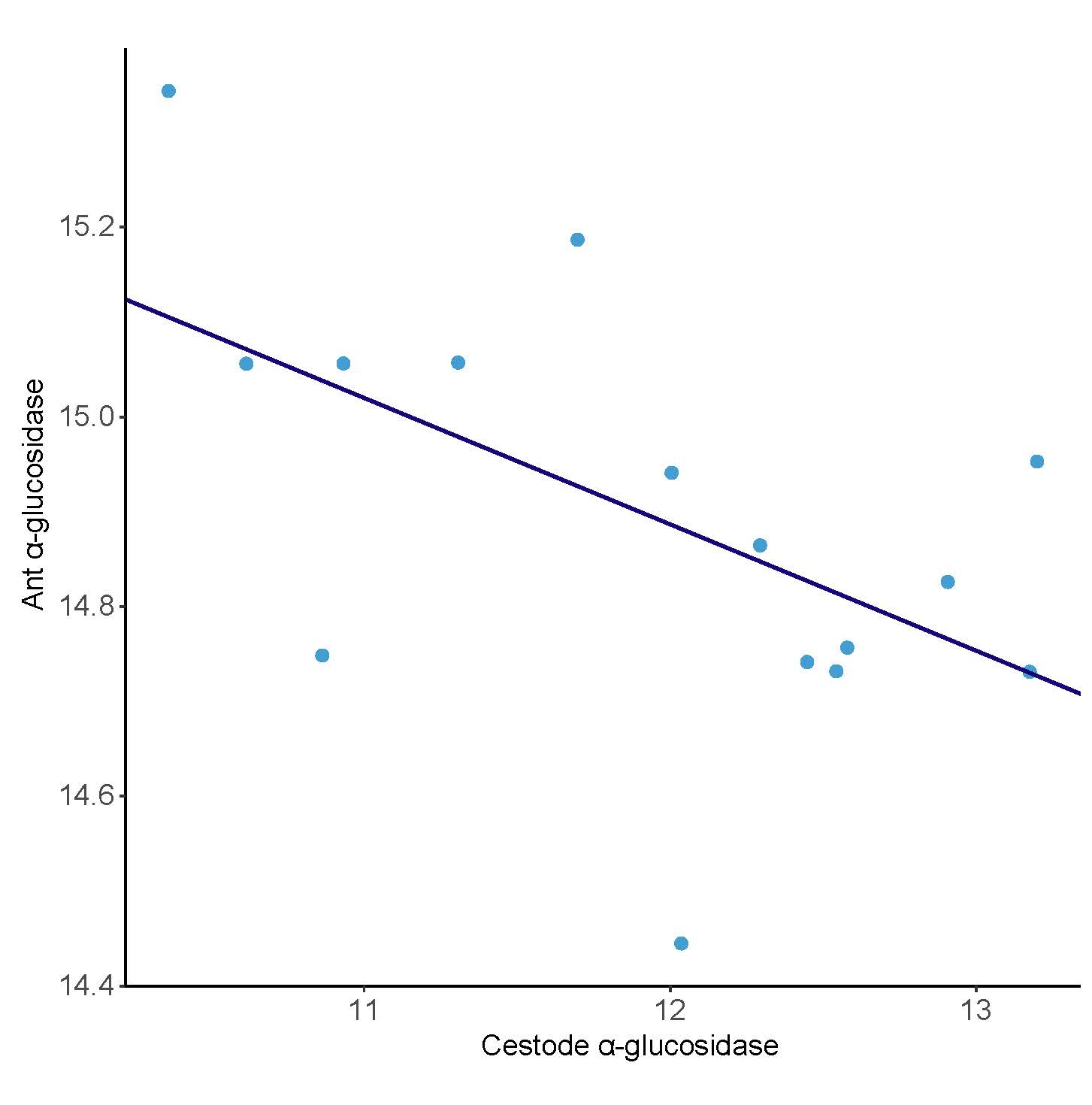


**Figure S2** Cestode alpha-glucosidase and ant alpha-glucosidase plotted against each other after variance stabilizing transformation. Here we found a negative correlation with an R value of -0.113 and an intercept of 16.5 (p=0.032).

**Table S1** overview of all the samples used in this paper with all information used for the analysis.

| **Colony** | **Infected sample name** | **Cestode sample name** | **Cestode number** | **Colony size** |
| --- | --- | --- | --- | --- |
| A | A6F | A6P | 8 | 77 |
| C | C1F | C1P | 5 | 44 |
| D | D5F | D5P | 7 | 164 |
| F | F2F | F2P | 8 | 65 |
| G | G1F | G1P | 8 | 93 |
| H | H1F | H1P | 10 | 129 |
| J | J8F | J8P | 7 | 72 |
| K | K4F | K4P | 6 | 137 |
| L | L18F | L18P | 7 | 177 |
| M | M16F | M16P | 6 | 121 |
| O | O1F | O1P | 7 | 86 |
| P | P2F | P2P | 5 | 16 |
| Q | Q4F | Q4P | 8 | 72 |
| S | S3F | S3P | 13 | 17 |
| T | T1F | T1P | 9 | 76 |

**Table S2** overview of all GO terms found for all annotated genes in the proteome

| **GO ID** | **Term** | **Annotated** | **Significant** | **Expected** | **Fisher** | **Gene name** |
| --- | --- | --- | --- | --- | --- | --- |
| GO:0014878 | response to electrical stimulus involved in regulation of muscle adaptation | 3 | 2 | 0.06 | 0.0013 | Superoxide dismutase |
| GO:0070262 | peptidyl-serine dephosphorylation | 7 | 2 | 0.15 | 0.0089 | Superoxide dismutase |
| GO:0007099 | centriole replication | 18 | 3 | 0.39 | 0.0061 | Superoxide dismutase |
| GO:0046718 | viral entry into host cell | 18 | 3 | 0.39 | 0.0061 | Superoxide dismutase |
| GO:0098655 | cation transmembrane transport | 200 | 10 | 4.29 | 0.0054 | Superoxide dismutase |
| GO:0045956 | positive regulation of calcium ion-dependent exocytosis | 7 | 2 | 0.08 | 0.0023 | Disulfide isomerase |
| GO:0007187 | G protein-coupled receptor signaling pathway, coupled to cyclic nucleotide second messenger | 32 | 3 | 0.35 | 0.0047 | Disulfide isomerase |
| GO:0007618 | mating | 69 | 4 | 0.75 | 0.0062 | Disulfide isomerase |
| GO:0015705 | iodide transport | 2 | 2 | 0.08 | 0.0017 | Thioredoxin peroxidase |
| GO:0014878 | response to electrical stimulus involved in regulation of muscle adaptation | 3 | 2 | 0.13 | 0.0051 | Thioredoxin peroxidase |
| GO:0015866 | ADP transport | 3 | 2 | 0.13 | 0.0051 | Thioredoxin peroxidase |
| GO:0015867 | ATP transport | 3 | 2 | 0.13 | 0.0051 | Thioredoxin peroxidase |
| GO:0001178 | regulation of transcriptional start site selection at RNA polymerase II promoter | 3 | 2 | 0.13 | 0.0051 | Thioredoxin peroxidase |
| GO:0035970 | peptidyl-threonine dephosphorylation | 4 | 2 | 0.17 | 0.0099 | Thioredoxin peroxidase |
| GO:1990253 | cellular response to leucine starvation | 4 | 2 | 0.17 | 0.0099 | Thioredoxin peroxidase |
| GO:0070262 | peptidyl-serine dephosphorylation | 7 | 4 | 0.29 | 0.0006 | Thioredoxin peroxidase |
| GO:0060078 | regulation of postsynaptic membrane potential | 36 | 4 | 1.5 | 0.0098 | Thioredoxin peroxidase |
| GO:0034329 | cell junction assembly | 210 | 9 | 8.78 | 0.0018 | Thioredoxin peroxidase |
| GO:0043161 | proteasome-mediated ubiquitin-dependent protein catabolic process | 187 | 12 | 7.82 | 0.009 | Thioredoxin peroxidase |
| GO:0040039 | inductive cell migration | 7 | 5 | 1.48 | 0.006 | Lysosomal alpha glucosidase |
| GO:0032876 | negative regulation of DNA endoreduplication | 7 | 5 | 1.48 | 0.006 | Lysosomal alpha glucosidase |
| GO:0006369 | termination of RNA polymerase II transcription | 9 | 6 | 1.91 | 0.0041 | Lysosomal alpha glucosidase |
| GO:0030206 | chondroitin sulfate biosynthetic process | 10 | 6 | 2.12 | 0.0085 | Lysosomal alpha glucosidase |
| GO:0048145 | regulation of fibroblast proliferation | 21 | 7 | 4.45 | 0.002 | Lysosomal alpha glucosidase |
| GO:1901222 | regulation of NIK/NF-kappaB signaling | 23 | 7 | 4.87 | 0.0095 | Lysosomal alpha glucosidase |
| GO:0008362 | chitin-based embryonic cuticle biosynthetic process | 11 | 8 | 2.33 | 0.0004 | Lysosomal alpha glucosidase |
| GO:0000729 | DNA double-strand break processing | 18 | 9 | 3.81 | 0.0084 | Lysosomal alpha glucosidase |
| GO:0007112 | male meiosis cytokinesis | 22 | 10 | 4.66 | 0.0091 | Lysosomal alpha glucosidase |
| GO:0060840 | artery development | 37 | 10 | 7.84 | 0.0095 | Lysosomal alpha glucosidase |
| GO:0046329 | negative regulation of JNK cascade | 22 | 10 | 4.66 | 0.0096 | Lysosomal alpha glucosidase |
| GO:0006970 | response to osmotic stress | 33 | 12 | 6.99 | 0.0017 | Lysosomal alpha glucosidase |
| GO:0032456 | endocytic recycling | 32 | 12 | 6.78 | 0.0061 | Lysosomal alpha glucosidase |
| GO:0043001 | Golgi to plasma membrane protein transport | 32 | 13 | 6.78 | 0.0096 | Lysosomal alpha glucosidase |
| GO:0007249 | I-kappaB kinase/NF-kappaB signaling | 43 | 14 | 9.11 | 0.0016 | Lysosomal alpha glucosidase |
| GO:0090307 | mitotic spindle assembly | 40 | 16 | 8.47 | 0.0057 | Lysosomal alpha glucosidase |
| GO:0051260 | protein homooligomerization | 81 | 23 | 17.15 | 0.0061 | Lysosomal alpha glucosidase |
| GO:0061025 | membrane fusion | 71 | 24 | 15.03 | 0.0082 | Lysosomal alpha glucosidase |
| GO:0044403 | biological process involved in symbiotic interaction | 131 | 35 | 27.74 | 0.0077 | Lysosomal alpha glucosidase |
| GO:0048608 | reproductive structure development | 184 | 39 | 38.96 | 0.0059 | Lysosomal alpha glucosidase |
| GO:0070201 | regulation of establishment of protein localization | 200 | 48 | 42.35 | 0.0093 | Lysosomal alpha glucosidase |
| GO:0031175 | neuron projection development | 494 | 106 | 104.61 | 0.0005 | Lysosomal alpha glucosidase |
| GO:0032268 | regulation of cellular protein metabolic process | 711 | 174 | 150.56 | 0.01 | Lysosomal alpha glucosidase |

**Table S3** overview of all GO terms found for all unannotated genes in the proteome

| **GO ID** | **Term** | **Annotated** | **Significant** | **Expected** | **Fisher** | **Ranking in haemolymph expression** |
| --- | --- | --- | --- | --- | --- | --- |
| GO:0035204 | negative regulation of lamellocyte differentiation | 7 | 2 | 0.06 | 0.0013 | 2nd most expressed |
| GO:0010613 | positive regulation of cardiac muscle hypertrophy | 15 | 2 | 0.12 | 0.0062 | 2nd most expressed |
| GO:0060395 | SMAD protein signal transduction | 18 | 2 | 0.15 | 0.0089 | 2nd most expressed |
| GO:0045956 | positive regulation of calcium ion-dependent exocytosis | 7 | 2 | 0.01 | <0.0001 | 3rd most expressed |
| GO:0071422 | succinate transmembrane transport | 2 | 2 | 0.06 | 0.0008 | 4th most expressed |
| GO:0042713 | sperm ejaculation | 3 | 2 | 0.09 | 0.0024 | 4th most expressed |
| GO:0010873 | positive regulation of cholesterol esterification | 3 | 2 | 0.09 | 0.0024 | 4th most expressed |
| GO:1903699 | tarsal gland development | 3 | 2 | 0.09 | 0.0024 | 4th most expressed |
| GO:0043651 | linoleic acid metabolic process | 3 | 2 | 0.09 | 0.0024 | 4th most expressed |
| GO:1903966 | monounsaturated fatty acid biosynthetic process | 3 | 2 | 0.09 | 0.0024 | 4th most expressed |
| GO:0006723 | cuticle hydrocarbon biosynthetic process | 3 | 2 | 0.09 | 0.0024 | 4th most expressed |
| GO:0010378 | temperature compensation of the circadian clock | 4 | 2 | 0.11 | 0.0047 | 4th most expressed |
| GO:0070474 | positive regulation of uterine smooth muscle contraction | 4 | 2 | 0.11 | 0.0047 | 4th most expressed |
| GO:0035338 | long-chain fatty-acyl-CoA biosynthetic process | 4 | 2 | 0.11 | 0.0047 | 4th most expressed |
| GO:0032025 | response to cobalt ion | 4 | 2 | 0.11 | 0.0047 | 4th most expressed |
| GO:0036109 | alpha-linolenic acid metabolic process | 4 | 2 | 0.11 | 0.0047 | 4th most expressed |
| GO:1904058 | positive regulation of sensory perception of pain | 4 | 2 | 0.11 | 0.0047 | 4th most expressed |
| GO:0048133 | male germ-line stem cell asymmetric division | 5 | 2 | 0.14 | 0.0077 | 4th most expressed |
| GO:0034625 | fatty acid elongation, monounsaturated fatty acid | 5 | 2 | 0.14 | 0.0077 | 4th most expressed |
| GO:0034626 | fatty acid elongation, polyunsaturated fatty acid | 5 | 2 | 0.14 | 0.0077 | 4th most expressed |
| GO:0019367 | fatty acid elongation, saturated fatty acid | 5 | 2 | 0.14 | 0.0077 | 4th most expressed |
| GO:0035844 | cloaca development | 5 | 2 | 0.14 | 0.0077 | 4th most expressed |
| GO:0006572 | tyrosine catabolic process | 5 | 3 | 0.14 | 0.0002 | 4th most expressed |
| GO:0006559 | L-phenylalanine catabolic process | 6 | 3 | 0.17 | 0.0004 | 4th most expressed |
| GO:0019432 | triglyceride biosynthetic process | 19 | 4 | 0.55 | 0.0017 | 4th most expressed |
| GO:0016318 | ommatidial rotation | 25 | 5 | 0.72 | 0.0006 | 4th most expressed |
| GO:0090175 | regulation of establishment of planar polarity | 43 | 5 | 1.23 | 0.0041 | 4th most expressed |
| GO:0046949 | fatty-acyl-CoA biosynthetic process | 12 | 6 | 0.34 | <0.0001 | 4th most expressed |
| GO:0006572 | tyrosine catabolic process | 5 | 3 | 0.03 | <0.0001 | 6th most expressed |
| GO:0006559 | L-phenylalanine catabolic process | 6 | 3 | 0.04 | <0.0001 | 6th most expressed |
| GO:0042438 | melanin biosynthetic process | 12 | 2 | 0.11 | 0.0048 | 12th most expressed |
| GO:0050884 | neuromuscular process controlling posture | 13 | 2 | 0.12 | 0.0057 | 12th most expressed |
| GO:0010613 | positive regulation of cardiac muscle hypertrophy | 15 | 2 | 0.13 | 0.0075 | 12th most expressed |
| GO:0007628 | adult walking behavior | 27 | 3 | 0.24 | 0.0016 | 12th most expressed |
| GO:0060395 | SMAD protein signal transduction | 18 | 2 | 0.09 | 0.0035 | 13th most expressed |

**Supplemental methodology**

As detailed in Sistermans et al. 2025 [13], colonies of *Temnothorax nylanderi* were gathered in the Lennebergwald in Mainz, Germany in late 2019 and early 2020. The ants relevant to our study were kept under standardized laboratory conditions and were dissected for their fat body and cestodes [13]. In total they yielded 15 infected ants and their corresponding parasites which are specified in table S1. Both parasite and host samples were placed in individual Eppendorf tubes containing 50 µL of trizol. RNA of these samples was extracted using the Qiagen RNeasy extraction kit. These samples were then sent to Novogene for RNA sequencing (for details regarding this procedure please be referred to Sistermans et al. 2025). Raw reads of *Anomotaenia brevis* were then mapped against their *A. brevis* genome combined with a *T. nylanderi* genome assembly [74] using STAR [75]. They then constructed a gene count matrix using htseq-count [76], filtering out all reads that mapped multiple times (filtering out potential contaminant reads). For *T. nylanderi* they first filtered raw reads against the genomes of *A. brevis*, humans and *E. coli* using fastqscreen [77] and then trimmed the reads using fastp [78]. Reads were then mapped against the earlier-mentioned *T. nylanderi* genome [74] using hisat [79] and created a gene count matrix as well as a genome-guided transcriptome assembly using stringtie [80]. BLAST annotation of both genomes was obtained using BLAST DIAMOND [81] and both GO and KEGG terms were obtained using eggNOG [82]. Besides raw reads (found on SRA, PRJNA1246159) all these data can be found on Dryad (DOI: 10.5061/dryad.8cz8w9h3b).
