# Supplementary material for: Integrated co-expression analysis of host–parasite transcriptomes reveals mechanisms of host modulation in an ant–cestode system
Source: BMC Genomics. 2026 Jan 31;27:232. doi: 10.1186/s12864-026-12581-6 (PMC12930657; doi:10.1186/s12864-026-12581-6)
Supplement: Supplementary file 2 — Supplementary Material 2. [file 12864_2026_12581_MOESM2_ESM.docx]

**Integrated Co-expression Analysis of Host–Parasite Transcriptomes Reveals Mechanisms of Host Modulation in an Ant–Cestode System**Tom Sistermans^1^, Romain Libbrecht^2^, Susanne Foitzik^1^

^1^Institute of Organismic and Molecular Evolution, Johannes Gutenberg University, Mainz, Germany

^2^Insect Biology Research Institute, UMR 7261, CNRS, University of Tours, France

**Supplementary materials**


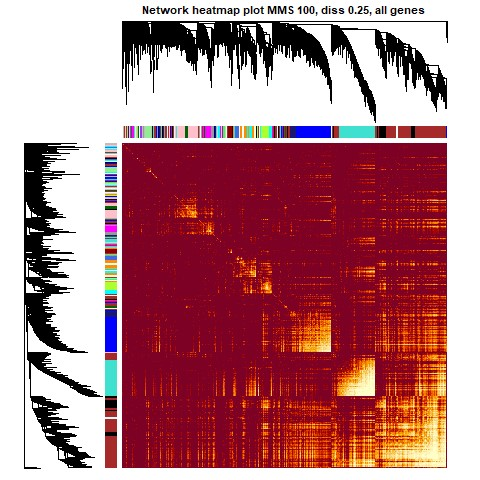


**Figure S1** TOM plot of the merged gene count matrices of both infected ants and their cestodes. The colours representing the constructed modules roughly aligning with the highest correlations in the heatmap.


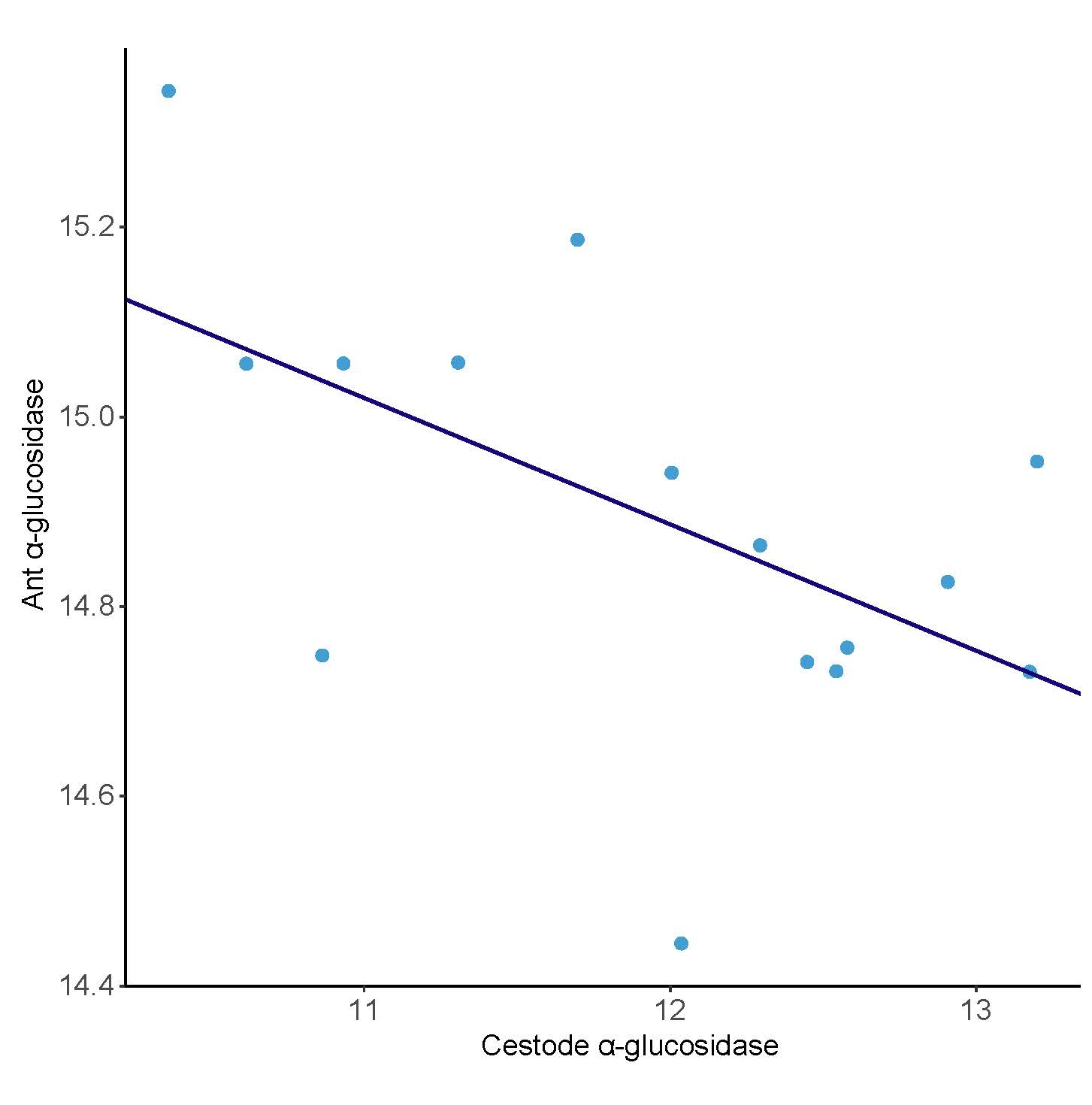


**Figure S2** Cestode alpha-glucosidase and ant alpha-glucosidase plotted against each other after variance stabilizing transformation. Here we found a negative correlation with an R value of -0.113 and an intercept of 16.5 (p=0.032).


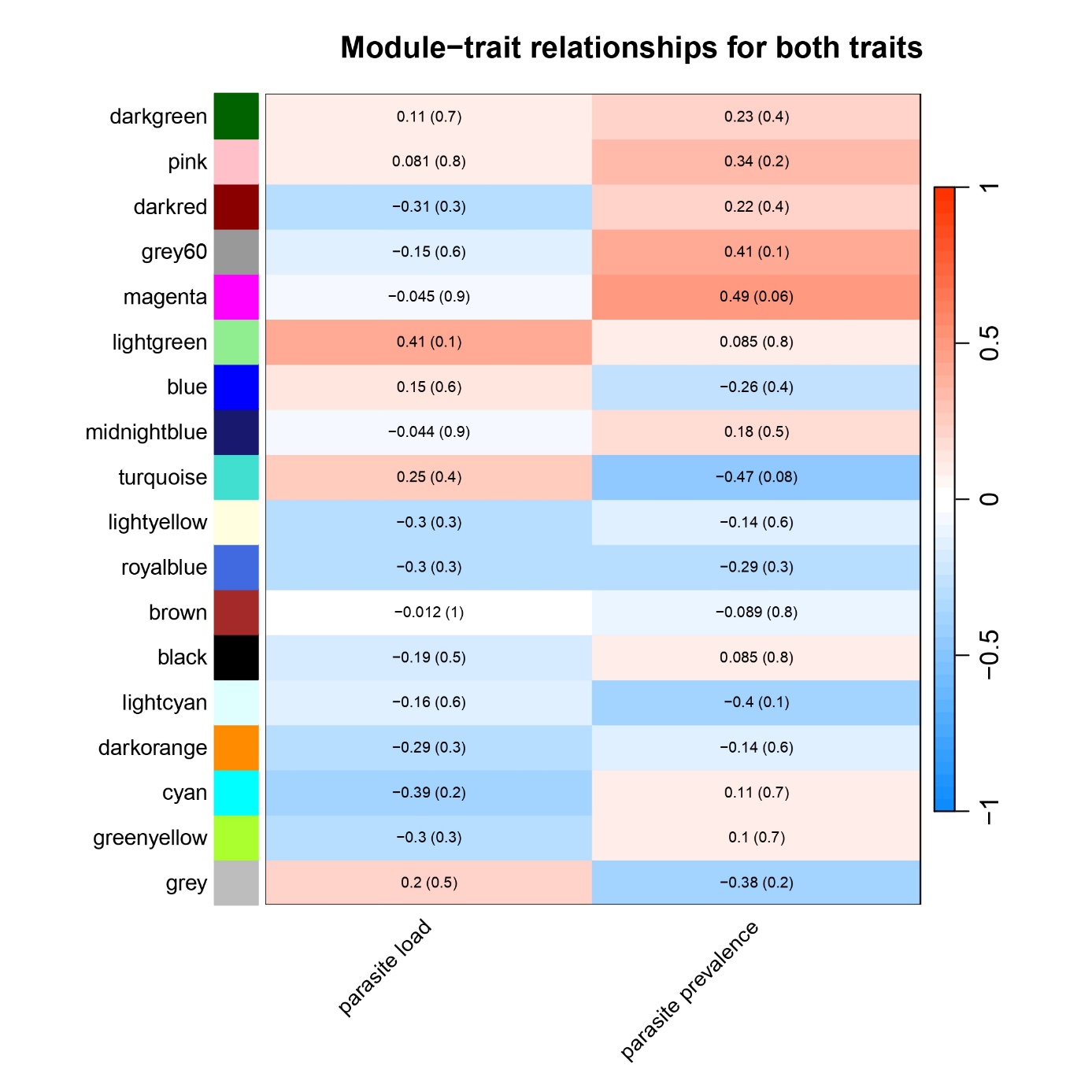


**Figure S3** Heatmap of correlations between module eigengene values and (left) parasite load or (right) parasite prevalence. Within every cell of the heatmap the correlation coefficient is mentioned and the P-value behind it between brackets.


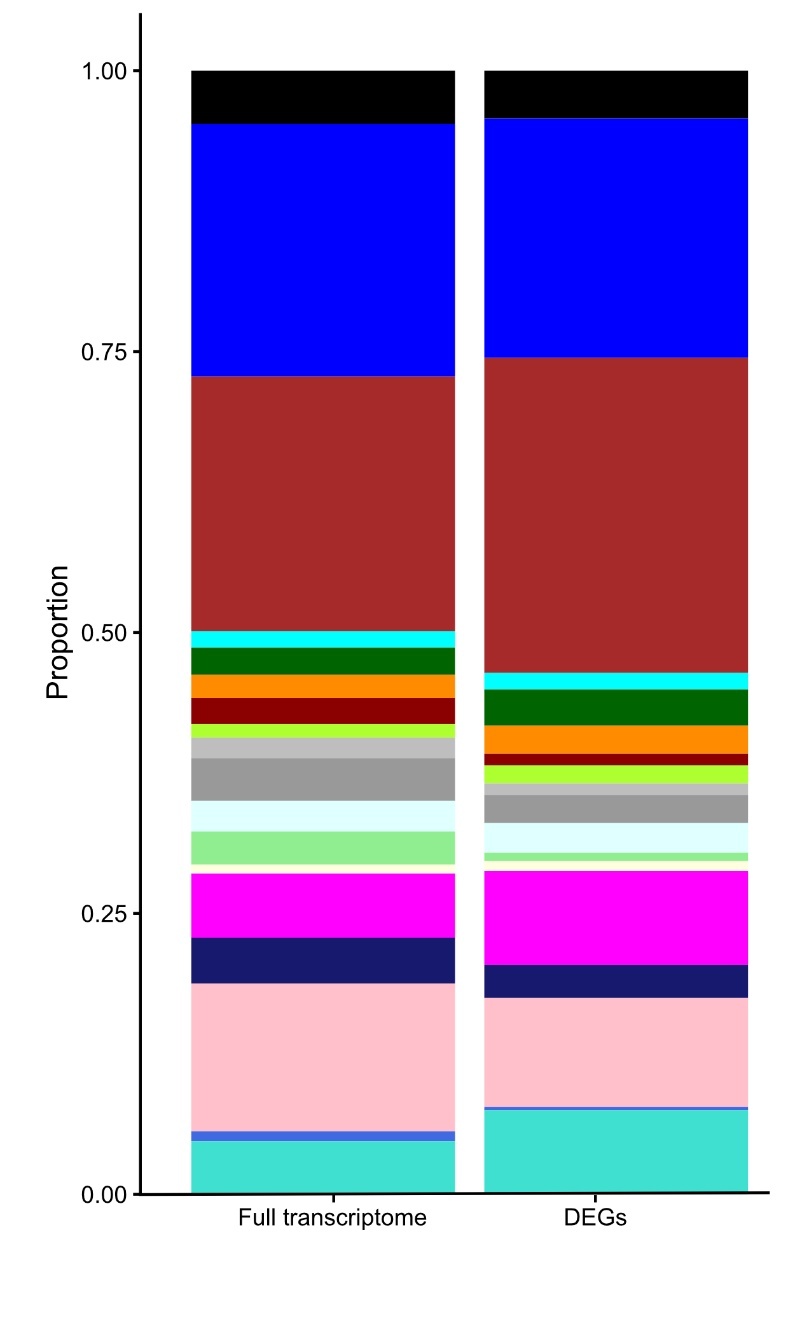


**Figure S4** Proportions of genes belonging to which module in (left) the full host transcriptome and (right) all DEGs upon cestode infection.


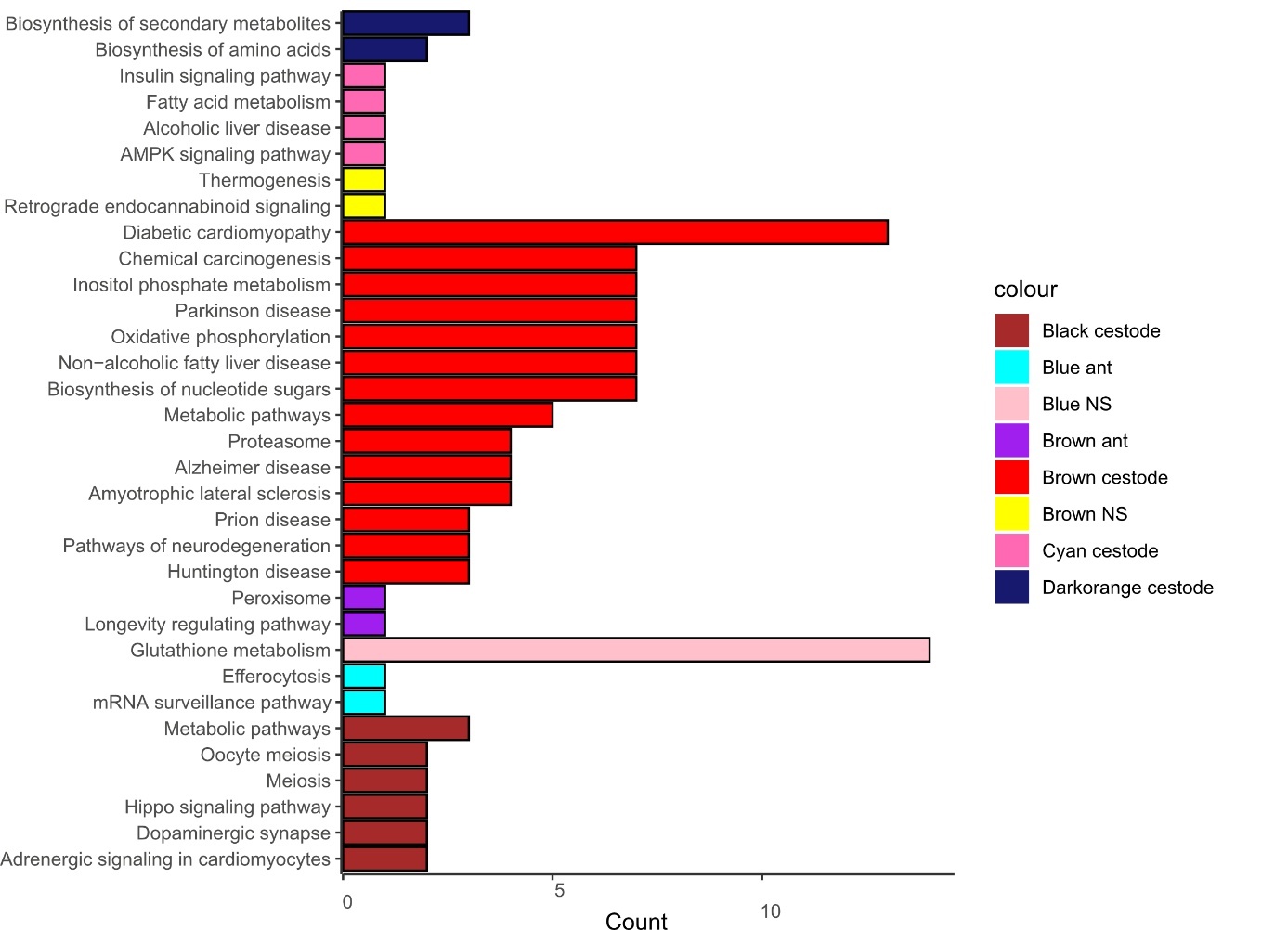


**Figure S5** KEGG enrichment of differentially expressed genes grouped by modules that correlate more strongly than expected by chance with cestode genes, ant genes, or show no significant correlation bias. Different colours indicate the respective groups.

**Table S1** overview of all the samples used in this paper with all information used for the analysis.

| **Colony** | **Infected sample name** | **Cestode sample name** | **Cestode number** | **Colony size** |
| --- | --- | --- | --- | --- |
| A | A6F | A6P | 8 | 77 |
| C | C1F | C1P | 5 | 44 |
| D | D5F | D5P | 7 | 164 |
| F | F2F | F2P | 8 | 65 |
| G | G1F | G1P | 8 | 93 |
| H | H1F | H1P | 10 | 129 |
| J | J8F | J8P | 7 | 72 |
| K | K4F | K4P | 6 | 137 |
| L | L18F | L18P | 7 | 177 |
| M | M16F | M16P | 6 | 121 |
| O | O1F | O1P | 7 | 86 |
| P | P2F | P2P | 5 | 16 |
| Q | Q4F | Q4P | 8 | 72 |
| S | S3F | S3P | 13 | 17 |
| T | T1F | T1P | 9 | 76 |

**Table S2** Overview of all GO terms found for all annotated genes in the proteome

| **GO ID** | **Term** | **Annotated** | **Significant** | **Expected** | **Fisher** | **Gene name** |
| --- | --- | --- | --- | --- | --- | --- |
| GO:0014878 | response to electrical stimulus involved in regulation of muscle adaptation | 3 | 2 | 0.06 | 0.0013 | Superoxide dismutase |
| GO:0070262 | peptidyl-serine dephosphorylation | 7 | 2 | 0.15 | 0.0089 | Superoxide dismutase |
| GO:0007099 | centriole replication | 18 | 3 | 0.39 | 0.0061 | Superoxide dismutase |
| GO:0046718 | viral entry into host cell | 18 | 3 | 0.39 | 0.0061 | Superoxide dismutase |
| GO:0098655 | cation transmembrane transport | 200 | 10 | 4.29 | 0.0054 | Superoxide dismutase |
| GO:0045956 | positive regulation of calcium ion-dependent exocytosis | 7 | 2 | 0.08 | 0.0023 | Disulfide isomerase |
| GO:0007187 | G protein-coupled receptor signaling pathway, coupled to cyclic nucleotide second messenger | 32 | 3 | 0.35 | 0.0047 | Disulfide isomerase |
| GO:0007618 | mating | 69 | 4 | 0.75 | 0.0062 | Disulfide isomerase |
| GO:0015705 | iodide transport | 2 | 2 | 0.08 | 0.0017 | Thioredoxin peroxidase |
| GO:0014878 | response to electrical stimulus involved in regulation of muscle adaptation | 3 | 2 | 0.13 | 0.0051 | Thioredoxin peroxidase |
| GO:0015866 | ADP transport | 3 | 2 | 0.13 | 0.0051 | Thioredoxin peroxidase |
| GO:0015867 | ATP transport | 3 | 2 | 0.13 | 0.0051 | Thioredoxin peroxidase |
| GO:0001178 | regulation of transcriptional start site selection at RNA polymerase II promoter | 3 | 2 | 0.13 | 0.0051 | Thioredoxin peroxidase |
| GO:0035970 | peptidyl-threonine dephosphorylation | 4 | 2 | 0.17 | 0.0099 | Thioredoxin peroxidase |
| GO:1990253 | cellular response to leucine starvation | 4 | 2 | 0.17 | 0.0099 | Thioredoxin peroxidase |
| GO:0070262 | peptidyl-serine dephosphorylation | 7 | 4 | 0.29 | 0.0006 | Thioredoxin peroxidase |
| GO:0060078 | regulation of postsynaptic membrane potential | 36 | 4 | 1.5 | 0.0098 | Thioredoxin peroxidase |
| GO:0034329 | cell junction assembly | 210 | 9 | 8.78 | 0.0018 | Thioredoxin peroxidase |
| GO:0043161 | proteasome-mediated ubiquitin-dependent protein catabolic process | 187 | 12 | 7.82 | 0.009 | Thioredoxin peroxidase |
| GO:0040039 | inductive cell migration | 7 | 5 | 1.48 | 0.006 | Lysosomal alpha glucosidase |
| GO:0032876 | negative regulation of DNA endoreduplication | 7 | 5 | 1.48 | 0.006 | Lysosomal alpha glucosidase |
| GO:0006369 | termination of RNA polymerase II transcription | 9 | 6 | 1.91 | 0.0041 | Lysosomal alpha glucosidase |
| GO:0030206 | chondroitin sulfate biosynthetic process | 10 | 6 | 2.12 | 0.0085 | Lysosomal alpha glucosidase |
| GO:0048145 | regulation of fibroblast proliferation | 21 | 7 | 4.45 | 0.002 | Lysosomal alpha glucosidase |
| GO:1901222 | regulation of NIK/NF-kappaB signaling | 23 | 7 | 4.87 | 0.0095 | Lysosomal alpha glucosidase |
| GO:0008362 | chitin-based embryonic cuticle biosynthetic process | 11 | 8 | 2.33 | 0.0004 | Lysosomal alpha glucosidase |
| GO:0000729 | DNA double-strand break processing | 18 | 9 | 3.81 | 0.0084 | Lysosomal alpha glucosidase |
| GO:0007112 | male meiosis cytokinesis | 22 | 10 | 4.66 | 0.0091 | Lysosomal alpha glucosidase |
| GO:0060840 | artery development | 37 | 10 | 7.84 | 0.0095 | Lysosomal alpha glucosidase |
| GO:0046329 | negative regulation of JNK cascade | 22 | 10 | 4.66 | 0.0096 | Lysosomal alpha glucosidase |
| GO:0006970 | response to osmotic stress | 33 | 12 | 6.99 | 0.0017 | Lysosomal alpha glucosidase |
| GO:0032456 | endocytic recycling | 32 | 12 | 6.78 | 0.0061 | Lysosomal alpha glucosidase |
| GO:0043001 | Golgi to plasma membrane protein transport | 32 | 13 | 6.78 | 0.0096 | Lysosomal alpha glucosidase |
| GO:0007249 | I-kappaB kinase/NF-kappaB signaling | 43 | 14 | 9.11 | 0.0016 | Lysosomal alpha glucosidase |
| GO:0090307 | mitotic spindle assembly | 40 | 16 | 8.47 | 0.0057 | Lysosomal alpha glucosidase |
| GO:0051260 | protein homooligomerization | 81 | 23 | 17.15 | 0.0061 | Lysosomal alpha glucosidase |
| GO:0061025 | membrane fusion | 71 | 24 | 15.03 | 0.0082 | Lysosomal alpha glucosidase |
| GO:0044403 | biological process involved in symbiotic interaction | 131 | 35 | 27.74 | 0.0077 | Lysosomal alpha glucosidase |
| GO:0048608 | reproductive structure development | 184 | 39 | 38.96 | 0.0059 | Lysosomal alpha glucosidase |
| GO:0070201 | regulation of establishment of protein localization | 200 | 48 | 42.35 | 0.0093 | Lysosomal alpha glucosidase |
| GO:0031175 | neuron projection development | 494 | 106 | 104.61 | 0.0005 | Lysosomal alpha glucosidase |
| GO:0032268 | regulation of cellular protein metabolic process | 711 | 174 | 150.56 | 0.01 | Lysosomal alpha glucosidase |

**Table S3** Overview of all GO terms found for all unannotated genes in the proteome

| **GO ID** | **Term** | **Annotated** | **Significant** | **Expected** | **Fisher** | **Ranking in haemolymph expression** |
| --- | --- | --- | --- | --- | --- | --- |
| GO:0035204 | negative regulation of lamellocyte differentiation | 7 | 2 | 0.06 | 0.0013 | 2nd most expressed |
| GO:0010613 | positive regulation of cardiac muscle hypertrophy | 15 | 2 | 0.12 | 0.0062 | 2nd most expressed |
| GO:0060395 | SMAD protein signal transduction | 18 | 2 | 0.15 | 0.0089 | 2nd most expressed |
| GO:0045956 | positive regulation of calcium ion-dependent exocytosis | 7 | 2 | 0.01 | <0.0001 | 3rd most expressed |
| GO:0071422 | succinate transmembrane transport | 2 | 2 | 0.06 | 0.0008 | 4th most expressed |
| GO:0042713 | sperm ejaculation | 3 | 2 | 0.09 | 0.0024 | 4th most expressed |
| GO:0010873 | positive regulation of cholesterol esterification | 3 | 2 | 0.09 | 0.0024 | 4th most expressed |
| GO:1903699 | tarsal gland development | 3 | 2 | 0.09 | 0.0024 | 4th most expressed |
| GO:0043651 | linoleic acid metabolic process | 3 | 2 | 0.09 | 0.0024 | 4th most expressed |
| GO:1903966 | monounsaturated fatty acid biosynthetic process | 3 | 2 | 0.09 | 0.0024 | 4th most expressed |
| GO:0006723 | cuticle hydrocarbon biosynthetic process | 3 | 2 | 0.09 | 0.0024 | 4th most expressed |
| GO:0010378 | temperature compensation of the circadian clock | 4 | 2 | 0.11 | 0.0047 | 4th most expressed |
| GO:0070474 | positive regulation of uterine smooth muscle contraction | 4 | 2 | 0.11 | 0.0047 | 4th most expressed |
| GO:0035338 | long-chain fatty-acyl-CoA biosynthetic process | 4 | 2 | 0.11 | 0.0047 | 4th most expressed |
| GO:0032025 | response to cobalt ion | 4 | 2 | 0.11 | 0.0047 | 4th most expressed |
| GO:0036109 | alpha-linolenic acid metabolic process | 4 | 2 | 0.11 | 0.0047 | 4th most expressed |
| GO:1904058 | positive regulation of sensory perception of pain | 4 | 2 | 0.11 | 0.0047 | 4th most expressed |
| GO:0048133 | male germ-line stem cell asymmetric division | 5 | 2 | 0.14 | 0.0077 | 4th most expressed |
| GO:0034625 | fatty acid elongation, monounsaturated fatty acid | 5 | 2 | 0.14 | 0.0077 | 4th most expressed |
| GO:0034626 | fatty acid elongation, polyunsaturated fatty acid | 5 | 2 | 0.14 | 0.0077 | 4th most expressed |
| GO:0019367 | fatty acid elongation, saturated fatty acid | 5 | 2 | 0.14 | 0.0077 | 4th most expressed |
| GO:0035844 | cloaca development | 5 | 2 | 0.14 | 0.0077 | 4th most expressed |
| GO:0006572 | tyrosine catabolic process | 5 | 3 | 0.14 | 0.0002 | 4th most expressed |
| GO:0006559 | L-phenylalanine catabolic process | 6 | 3 | 0.17 | 0.0004 | 4th most expressed |
| GO:0019432 | triglyceride biosynthetic process | 19 | 4 | 0.55 | 0.0017 | 4th most expressed |
| GO:0016318 | ommatidial rotation | 25 | 5 | 0.72 | 0.0006 | 4th most expressed |
| GO:0090175 | regulation of establishment of planar polarity | 43 | 5 | 1.23 | 0.0041 | 4th most expressed |
| GO:0046949 | fatty-acyl-CoA biosynthetic process | 12 | 6 | 0.34 | <0.0001 | 4th most expressed |
| GO:0006572 | tyrosine catabolic process | 5 | 3 | 0.03 | <0.0001 | 6th most expressed |
| GO:0006559 | L-phenylalanine catabolic process | 6 | 3 | 0.04 | <0.0001 | 6th most expressed |
| GO:0042438 | melanin biosynthetic process | 12 | 2 | 0.11 | 0.0048 | 12th most expressed |
| GO:0050884 | neuromuscular process controlling posture | 13 | 2 | 0.12 | 0.0057 | 12th most expressed |
| GO:0010613 | positive regulation of cardiac muscle hypertrophy | 15 | 2 | 0.13 | 0.0075 | 12th most expressed |
| GO:0007628 | adult walking behavior | 27 | 3 | 0.24 | 0.0016 | 12th most expressed |
| GO:0060395 | SMAD protein signal transduction | 18 | 2 | 0.09 | 0.0035 | 13th most expressed |

**Table S4** Numbers of differentially expressed genes upon parasite infection for each module and whether they are more strongly associated with parasite genes or host genes.

| **Module** | **Cestode** | **Ant** | **Not significant** |
| --- | --- | --- | --- |
| **Black** | 28 | 0 | 1 |
| **Blue** | 0 | 5 | 140 |
| **Brown** | 181 | 9 | 1 |
| **Cyan** | 10 | 0 | 0 |
| **Darkgreen** | 0 | 0 | 22 |
| **Darkorange** | 16 | 0 | 1 |
| **Darkred** | 1 | 2 | 4 |
| **Greenyellow** | 10 | 1 | 0 |
| **Grey** | 1 | 4 | 2 |
| **Grey60** | 0 | 0 | 17 |
| **Lightcyan** | 17 | 1 | 0 |
| **Lightgreen** | 0 | 4 | 1 |
| **Lightyellow** | 6 | 0 | 0 |
| **Magenta** | 0 | 0 | 57 |
| **Midnightblue** | 0 | 0 | 20 |
| **Pink** | 0 | 24 | 42 |
| **Royalblue** | 2 | 0 | 0 |
| **Turquoise** | 48 | 2 | 1 |
| **Total** | 320 | 52 | 309 |

**Methodological details**

As detailed in Sistermans et al. 2025 [1], colonies of *Temnothorax nylanderi* were gathered in the Lennebergwald in Mainz, Germany in late 2019 and early 2020. The ants relevant to our study were kept under standardized laboratory conditions and were dissected for their fat body and cestodes [1]. In total they yielded 15 infected ants and their corresponding parasites which are specified in table S1. Both parasite and host samples were placed in individual Eppendorf tubes containing 50 µL of trizol. RNA of these samples was extracted using the Qiagen RNeasy extraction kit. These samples were then sent to Novogene for RNA sequencing (for details regarding this procedure please be referred to Sistermans et al. 2025). Raw reads of *Anomotaenia brevis* were then mapped against their *A. brevis* genome combined with a *T. nylanderi* genome assembly [2] using STAR (version 2.7.10b) [3]. They then constructed a gene count matrix using htseq-count [4], filtering out all reads that mapped multiple times (filtering out potential contaminant reads). For *T. nylanderi* they first filtered raw reads against the genomes of *A. brevis*, humans and *E. coli* using fastqscreen (version 0.14.0) [5] and then trimmed the reads using fastp [6]. Reads were then mapped against the earlier-mentioned *T. nylanderi* genome [2] using hisat (version 2.1.0) [7] and created a gene count matrix as well as a genome-guided transcriptome assembly using stringtie (version 1.3.6) [8]. BLAST annotation of both genomes was obtained using BLAST DIAMOND [9] and both GO and KEGG terms were obtained using eggNOG [10]. Besides raw reads (found on SRA, PRJNA1246159) all these data can be found on Dryad (DOI: 10.5061/dryad.8cz8w9h3b).

**Links to genes differentially expressed between infected and uninfected workers**

We tested whether differentially expressed genes (DEGs) between infected and uninfected ants using DESeq2 (version 1.50.2; Love et al. 2014). We ran a Likelihood Ratio Test (LRT), testing parasite infection on the 15 infected and 15 uninfected samples taking the colony identity as reduced factor. Note that, despite having more metadata, like cestode number, colony size, and colony infection rate, we only took the colony identity as reduced factor since cestode number is heavily nested in infection, and colony size and infection rate are completely nested in colony identity. We then assigned a module colour to each of the DEGs and tested whether they were significantly more present than ant genes in the WGCNA module than should be expected by chance using a fisher’s exact test, correcting for multiple testing using the Benjamini Hochberg method. We further analysed whether DEGs were more likely to be hub genes through a Fisher’s exact test. We then tested whether DEGs correlated more with cestode genes or ant genes than should be expected by chance. We did this by calculating confidence intervals for the proportion of cestode genes in each module, due to the number of tests we would be doing, we Bonferroni-corrected our Z-scores by dividing the alpha by the number of tests. We then checked if the proportion of cestode genes each DEG correlates with is within the upper and lower bounds of the confidence interval, when it’s lower than the confidence interval, it means that the gene correlates significantly more with ant genes and when it’s higher, the gene correlates more with cestode genes. We then counted for each module how may genes correlate with every group. Finally, with the DEGs now grouped into modules and whether they are more strongly linked to parasite genes, host genes or non-significant based on module proportions we enriched KEGG terms for these groups of DEGs using the clusterProfiler R package (version 4.18.3) to untangle the possible functions of these genes.

We found a total of 683 DEGs for parasite infection, 368 of which were overexpressed in uninfected ants and 315 were overexpressed in infected ants. We then assigned to each gene their respective module and upon running Fisher’s exact test we found four modules with an adjusted P-value under 0.05, these being magenta, pink, lightgreen and turquoise. As we visually confirm (figure S4), DEGs had a significantly stronger presence in the turquoise and magenta modules whereas they were less strongly present in lightgreen and pink. This would suggest that the turquoise and magenta modules are more strongly influenced by the presence of cestode genes in our WGCNA dataset. However, given that all modules are represented in the DEGs, we believe it to be probable that our methods do not have a strong bias towards any one module.

When testing whether DEGs were more likely to be hub genes we employed Fisher’s exact test and found out that DEGs were not more likely to be hub genes (p-value=0.088). When we tested whether DEGs were more likely to correlate with cestode genes, we found strong evidence that this is the case (table S4), we found a total of 320 genes to be more strongly associated with cestode genes against a total of 52 genes that were more strongly associated with ant genes and 309 genes that did not deviate from the null-hypothesis.

Finally, we performed KEGG enrichment on DEG clusters defined by module membership and by whether genes correlated more strongly with cestode genes, ant genes, or showed no significant bias, as described above. KEGG terms were enriched for five modules (Fig. S5). Two modules showed enrichment across multiple correlation groups: blue (ant-correlated and non-significant genes) and brown (all three groups). DEGs in the brown module that correlated strongly with parasite genes were enriched for several neurodegenerative and neurological disease pathways, including *Prion disease* (ko05020), *Parkinson disease* (ko05012), *Alzheimer disease* (ko05010), and *Huntington disease* (ko05016). Additional cestode-correlated clusters were enriched for metabolic pathways, such as biosynthesis of secondary metabolites in the dark orange module.

**Link of parasite load and parasite prevalence to module eigenvalues**

Because our dataset includes variation in parasite prevalence and controlled differences in parasite load, both of which are important drivers of gene expression in this system, we tested whether either factor influenced module-level expression. For each module, we extracted eigengene values per sample and correlated them with parasite load and parasite prevalence using Pearson correlation tests.

We detected no significant correlations between module eigengenes and parasite load or parasite prevalence (Fig. S3). This was expected for parasite load, as it was controlled for in the original dataset. For parasite prevalence, any effects may be restricted to the differentially expressed genes identified previously, which may be insufficient to influence these much larger modules.

**References in supplement**

1. Sistermans T, Darras H, Rognet V, Beros S, Hartke J, Stoldt M, et al. Parasite prevalence in a social host has colony-wide impacts on transcriptional activity and survival. Evolution. 2025;:qpaf118. 10.1093/evolut/qpaf118.

2. Jongepier E, Séguret A, Labutin A, Feldmeyer B, Gstöttl C, Foitzik S, et al. Convergent Loss of Chemoreceptors across Independent Origins of Slave-Making in Ants. Molecular Biology and Evolution. 2022;39. 10.1093/molbev/msab305.

3. Dobin A, Davis CA, Schlesinger F, Drenkow J, Zaleski C, Jha S, et al. STAR: ultrafast universal RNA-seq aligner. Bioinformatics. 2013;29:15–21. 10.1093/bioinformatics/bts635.

4. Zanini F, Anders S, Pyl PT, Pimanda JE. Gene expression Analysing high-throughput sequencing data in Python with HTSeq 2.0. 2022;38 March:2943–5.

5. Wingett SW, Andrews S. Fastq screen: A tool for multi-genome mapping and quality control. F1000Research. 2018;7 May:1–13. 10.12688/f1000research.15931.1.

6. Chen S, Zhou Y, Chen Y, Gu J. Fastp: An ultra-fast all-in-one FASTQ preprocessor. Bioinformatics. 2018;34:i884–90. 10.1093/bioinformatics/bty560.

7. Kim D, Langmead B, Salzberg SL. HISAT: A fast spliced aligner with low memory requirements. Nature Methods. 2015;12:357–60. 10.1038/nmeth.3317.

8. Pertea M, Pertea GM, Antonescu CM, Chang TC, Mendell JT, Salzberg SL. StringTie enables improved reconstruction of a transcriptome from RNA-seq reads. Nature Biotechnology. 2015;33:290–5. 10.1038/nbt.3122.

9. Buchfink B, Xie C, Huson DH. Fast and sensitive protein alignment using DIAMOND. Nature Methods. 2014;12:59–63. 10.1038/nmeth.3176.

10. Cantalapiedra CP, Hern̗andez-Plaza A, Letunic I, Bork P, Huerta-Cepas J. eggNOG-mapper v2: Functional Annotation, Orthology Assignments, and Domain Prediction at the Metagenomic Scale. Molecular Biology and Evolution. 2021;38:5825–9. 10.1093/molbev/msab293.
